# Deep Conservation of *Hid*-Like RHG Gene Family Homologs in Winged Insects Revealed by “Taxon Hopping” BLAST

**DOI:** 10.3390/insects12110957

**Published:** 2021-10-21

**Authors:** Markus Friedrich

**Affiliations:** 1 Department of Biological Sciences, Wayne State University, 5047 Gullen Mall, Detroit, MI 48202, USA; friedrichm@wayne.edu; Tel.: +1-313-577-9612; Fax: +1-313-577-6891; 2 Department of Ophthalmological, Visual and Anatomical Sciences, School of Medicine, Wayne State University, 540 East Canfield Avenue, Detroit, MI 48202, USA

**Keywords:** *hid*, apoptosis, programmed cell death, *Drosophila*, Tenebrionidae, pea aphid, differential splicing, gene family evolution, taxon hopping BLAST

## Abstract

**Simple Summary:**

Programmed cell death is a universal mechanism in animal development and tissue maintenance, which facilitates the elimination of surplus or poorly functioning cells. Many conserved regulators of programmed cell death have been identified in model organisms including the fruit fly Drosophila melanogaster. In the latter, the four members of the RHG gene family function as critical inducers of programmed cell death. Despite this important role, RHG genes had thus far only been found in a surprisingly small number of insect groups, i.e., other flies and butterflies. This study reports the much deeper conservation of RHG genes in winged insects, ranging from cockroaches to beetles. In addition to opening new opportunities to study programmed cell death in a wide range of insects, the bioinformatic search strategy developed for this work will be of general use for studying gene families with challenging sequence evolution dynamics.

**Abstract:**

Together with *sickle* (*skl*), the *Drosophila* paralogs *reaper* (*rpr*), *head involution defective* (*hid*), and *grim* (RHG) control a critical switch in the induction of programmed cell death. RHG homologs have been identified in other dipteran and lepidopteran species but not beyond. Revisiting this issue with a “taxon hopping” BLAST search strategy in current genome and transcriptome resources, I detected high confidence RHG homologs in Coleoptera, Hymenoptera, Hemiptera, and Dictyoptera. Analyses of gene structure and protein sequence conservation revealed aconserved splicing pattern and highly conserved amino acid residues at both the N- and C-terminal ends that identify *hid* as the most ancestrally organized RHG gene family member in *Drosophila*. *hid*-like RHG homologs were also detected in mosquitoes, redefining their *michelob_x* (*mx*) genes as an expansion of derived RHG homologs. Only singleton homologs were detected in the large majority of other insect clades. Lepidopteran RHG homologs, however, stand out by producing an evolutionarily-derived splice isoform, identified in previous work, in addition to the newly detected *hid*-like isoform. Exceptional sequence diversification of select RHG homologs at the family- and genus-level explain their previous elusiveness in important insect genome model species like the red flour beetle *Tribolium castaneum* and the pea aphid *Acyrthosiphon pisum*. Combined, these findings expand the minimal age of the RHG gene family by about 100 million years and open new avenues for molecular cell death studies in insects.

## 1. Introduction

Programmed cell death results from the unleashed activity of caspases, a deeply conserved gene family of cysteinyl aspartate proteases. First characterized for their executive role in programmed cell death in the nematode *Caenorhabditis elegans* [1], subsequent studies in other model organisms, i.e., *Drosophila* and mice, uncovered the functional conservation of caspases as executive forces in the programmed cell death pathway [2]. However, the processes in control of appropriate caspase activation have been found to involve both conserved and diverged mechanisms. In mammals, for instance, mitochondrial signals and members of the Bcl2 gene family are in control of caspase activation [3]. In *C. elegans*, a similar, but less complex regulatory protein machinery appears to be in place [4]. In *Drosophila*, caspases are constitutively expressed but blocked by default through physical interventions by members of the inhibitor of apoptosis (IAP) gene family [5]. Pending developmental cues or cellular stress conditions, this block is relieved by the small protein products of the RHG gene family [6], which includes the name-giving paralogs *reaper* (*rpr*), *head involution defective* (*hid*), and *grim*, besides *sickle* (*skl*) [7,8].

*Rpr* was the first characterized *Drosophila* RHG gene family member [7], followed by *hid* [8], *grim* [9], and *skl* [10,11,12]. Subsequent efforts of identifying homologs in newly available *Drosophila* species genome drafts revealed the conservation of all four clustered genes in the Drosophilidae [13]. Similar efforts to find RHG homologs in the first genome draft of the Malaria vector mosquito species *Anopheles gambiae*, however, were unsuccessful [13]. At the same time, the comparative analysis of *Drosophila* RHG homologs corroborated the high conservation of the N-terminal IBM (IAP-binding motif) sequence [14,15]: A-[KTVI]-[PAE]-[FEISY]. This finding was consistent with the subsequent discovery that the inhibitory binding of RHG homologs to IAP proteins was dependent on the N-terminal amino acid residues [16]. Comparative sequence analyses further suggested the existence of IBM subtypes [13] and the presence of a second, putatively shared motif called Trp-block or Grim Helix 3 [17,18]. This progress notwithstanding, the challenge of identifying RHG genes on the basis of very limited sequence conservation culminated in the cautionary statement that even the relatedness of the *Drosophila* paralogs was only tentatively supported by sequence similarity [13].

Today, caspase and IAP genes have been identified in a wide range of insect species [19], but the search for RHG homologs has thus far been only successful in dipteran and lepidopteran species [20]. In Diptera, RHG homologs have been analyzed in the blowfly species *Lucilia cuprina* and *L. sericata* [21,22], the Caribbean fruit fly species *Anastrepha suspensa* (Tephritidae) [23], and, most recently, the scuttle fly *Megaselia scalaris* [20]. In the Lepidoptera, RHG homologs have been studied in the silkmoth *Bombyx mori* and the fall armyworm *Spodoptera frugiperda* [24,25,26]. The existence of RHG homologs outside the Lepidoptera and Diptera, however, has remained elusive. While it is possible that the RHG gene family originated in the lineage to the last common ancestor of the Lepidoptera and Diptera, which are relatively closely related insect orders [27] the short sequence lengths and low sequence conservation of RHG genes, however, are suspected to limit the detectability of distantly related homologs [13,19]. This challenge is exacerbated by the scarcity of cell death pathway studies in other insect models [19,28,29]. Here I report the results from searching current genome and transcriptome databases with a taxon hopping strategy that recovered RHG homologs from a substantially wider range of winged insect orders.

## 2. Results

### 2.1. RHG Homologs from an Extended Range of Winged Insects

Initial searches for RHG homologs outside Diptera and Lepidoptera were conducted using the silkworm RHG homolog *IAP-binding motif 1* (*IBM1*) (NP_001159813.1) as a query in BLASTp searches against the NCBI nr database (accessed on 1–31 December 2020) with Diptera and Lepidoptera excluded from the taxonomic search range [24]. This effort yielded low confidence hits against candidate homologs in the hemipteran species *Bemisia tabaci* (LOC109029550; e-value = 0.021), *Nilaparvata lugens* (LOC111048366; e-value = 0.005), and *Laodelphax striatellus* (RZF36208.1; 0.005). All of these sequences started with the RHG homology-defining IAP-binding motif (IBM) [13], were less than 300 amino acids long, and returned IBM1 as the single best hit when reBLASTed against the silkworm protein sequence database.

As the presence of RHG homologs in hemipteran species predicted the conservation of the RHG family throughout the Holometabola, I used the newly identified hemipteran sequences as queries in clade-specific BLAST searches for homologs in the Coleoptera (beetles) and Hymenoptera (bees + wasps). This approach produced significant hits in over 50 hymenopteran species, 21 of which were compiled for detailed analysis (Supplementary data file S1), and five coleopteran species. Among the latter, a notable absence was that of the flour beetle *Tribolium castaneum*, which represents the first coleopteran genome draft that has since been improved by a number of revisions [30,31]. I therefore continued to BLAST search for additional coleopteran RHG homologs using the newly detected coleopteran homologs as seed queries. One of them, i.e., the putative RHG homolog of the Emerald ash borer *Agrilus planipennis* (XP_018330969.1), detected the protein product of *T. castaneum* locus LOC103313285 (XP_008194456.1) as a candidate RHG homolog with an e-value of 0.05. ReBLAST of the *T. castaneum* LOC103313285 protein sequence against the conceptual *A. planipennis* proteome returned the putative *A. planipennis* RHG homolog as the best matching hit. Using the putative *T. castaneum* RHG homolog as a query against coleopteran transcript and protein sequence databases expanded the compilation of coleopteran RHG sequences to 25 (Supplementary data file S1). This included two further darkling beetle family homologs (*Asbolus verrucosus* and *Zophobas atratus*) and five additional homologs from families in the Tenebrionoidea (Supplementary data file S1).

**Figure 1:**
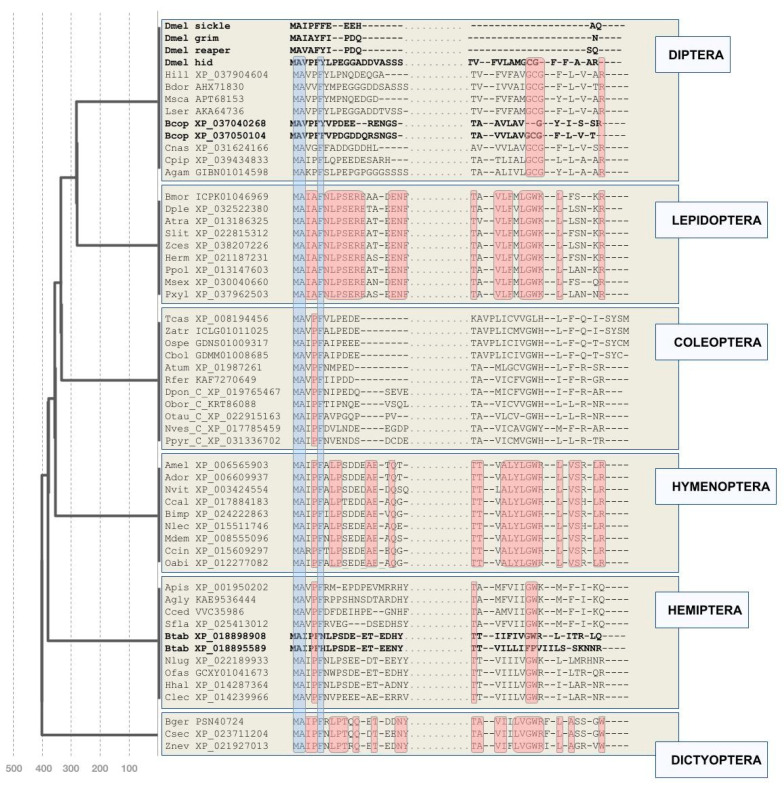
Overview on newly detected RHG homologs. Clustal Omega multiple sequence alignment of N- and C-terminal regions for a selection of newly RHG identified homologs. Blue overcasts: Residues conserved across all homologs. Red overcasts: Residues conserved across all homologs within orders (except within-species paralogs). Duplicated homologs in *D. melanogaster* (*sickle, grim, reaper, hid*), the fungus gnat *Bradysia odoriphaga* (Brad), and *Bemisia tabaci* (Btab) indicated by bold font. See Supplementary data file S1 for species name abbreviations. Numbers at the bottom of hatched vertical time lines correspond to millions of years past present time. Divergence time points based on [27].

Similar “taxon hopping” BLAST searches unearthed high confidence RHG homologs in a total of 19 hemipteran species including aphids as well as in three representatives of the Dictyoptera: The German cockroach *Blattella germanica* (PSN40724) and the termite species *Cryptotermes secundus* and *Zootermopsis nevadensis* (Figure 1). Extensive searches in further pancrustacean and invertebrate databases did not return candidate RHG homologs.

Most of the newly detected homologs outside the genus *Drosophila* were singletons except for duplicate pairs in the silverleaf whitefly *Bemisia tabaci* and the fungus gnat *Bradysia odoriphaga* (Figure 1), and the exceptional expansion of RHG homologs in mosquitoes (see below).

### 2.2. Protein Sequence Conservation Differences within and between Orders

The crucial success of “taxon hopping” in the detection of new RHG homologs constituted preliminary evidence of potentially different rates of RHG sequence change between and within insect orders. This idea was further supported by the clade-specific differences of sequence divergence in the most conserved protein sequence regions of the newly detected RHG homologs, i.e., the N- and C-terminal ends (Figure 1). To test for this possibility in a quantitative manner, I generated estimates of RHG protein sequence change rates within insect orders by determining average numbers of non-conserved sites in Clustal Omega multiple sequence alignments (MSAs) divided by respective clade ages (Table 1). By this measure, RHG protein sequence change rates varied up to more than 15-fold between select clades. The lowest rate was found for the hymenopteran RHG homologs with 0.18% per million years, while aphids stood out with the highest rate of close to 3% per million years (Table 1). These outliers excluded, the average RHG protein sequence change rate amounted to 0.34% (+/−0.07) per million years. More notable was the fact that the aphid protein sequence change rate of 3% per million years compared to 0.29% in the remaining hemipteran species sampled (Table 1). Thus, while approximate, these quantitative findings confirmed the existence of RHG protein sequence change rate differences between and within insect orders.

### 2.3. Deeply Conserved N- and C-Terminal Amino Acid Residues

Despite the partly dramatic differences in protein sequence divergence, MSAs of the newly compiled RHG protein sequences also identified deeply conserved amino acid residues. This was not only the case for the previously noted conserved N-terminal IBM but also for residues at the C-terminal end (Figure 1). Most consistent was the deployment of arginine (R) as the terminal amino acid residue, which is also the case for *Drosophila* RHG homolog *hid* (Figure 1). Besides the *Drosophila* RHG paralogs *skl*, *grim*, and *rpr*, further exceptions included the duplicated RHG homologs of *B. tabaci* and *B. odoriphaga* (Figure 1). Moreover, in all of the compiled aphid homologs, the ancestral C-terminal arginine residue was replaced by glutamine (Q). This feature was also shared by one of the duplicated homologs in the closely related *B. tabaci* (Figure 1). Further examples of clade-specific departure from the apparent C-terminal amino acid residue consensus included the exceptionally diverged C-termini in a subgroup of darkling beetles that included *T. castaneum* and in the three dictyopteran species, which shared a C-terminal tryptophan (W) residue (Figure 1).

The second-most consistently conserved C-terminal pattern was the combination of a glycine (G) residue followed by tryptophan (W) 5–7 residues away from the C-terminus in all clades except Diptera (Figure 1). The latter shared the conserved glycine residue, but the adjacent consensus tryptophan was replaced by a cysteine (C). Further exceptions from the GW consensus included the RHG homolog of *T. castaneum* and one of the two *B. tabaci* paralogs, XP_018895589, which lacked both residues (Figure 1).

There was also tentative evidence of protein sequence conservation further N-terminal from the conserved glycine–tryptophan duplet, which was more unambiguously documented in the sequence comparisons within orders than between orders (Figure 1). Overall, these findings unearthed evidence of conserved constraints at the C-terminal end of RHG proteins in addition to the N-terminal IBM. Moreover, these findings also defined *hid* as the most ancestrally organized RHG paralog in *Drosophila* given the complete lack of C-terminal consensus residues in *rpr*, *grim*, and *skl* (Figure 1).

### 2.4. Michelob_x Constitutes an Independent RHG Gene Family Expansion in Mosquitoes

The first RHG homologs outside the genus *Drosophila* were discovered in mosquitoes [32]. Completion of the genome sequence project of *A. gambiae* revealed the presence of conserved caspase and IAP genes, but the existence of RHG homologs had initially remained elusive [33,34]. Developing a hidden Markov model search profile for the RHG IBM motif from sequence comparisons of distantly related *Drosophila* species, Zhou et al. (2005) detected candidate RHG homologs in *A. gambiae*. One of these, named *michelob_x* (*mx*), was studied in detail and found to induce apoptosis in cell culture as well as transgenic *Drosophila*. Moreover, *A. gambiae* Mx was shown to bind *Drosophila* Diap1 in vitro and in an IBM-dependent manner [32].

**Figure 2:**
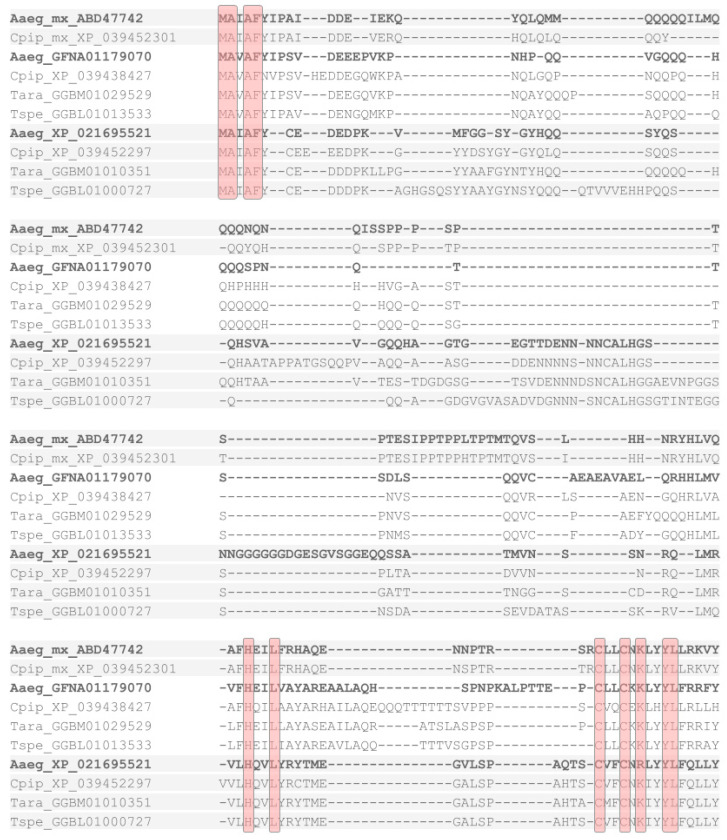
Multiple sequence alignment of mosquito *mx* paralogs. Multiple sequence alignment of *mx* homologs detected in *Aedes aegypti* (Aaeg), *Culex pipiens* (Cpip), *Tripteroides aranoides* (Tara), and *Toxorhynchites* spec. (Tspe). Residues conserved across all homologs highlighted by red overcast. *A. aegypti* homologs are highlighted in bold font for orientation.

Given the apparent lack of *hid*-like C-terminal consensus amino acid residues in mosquito *mx* homologs (Figure 2) [32], I conducted BLAST searches against mosquito genome and transcript databases with both *mx* and dipteran *hid*-like RHG homologs as queries. These efforts revealed the presence of *hid*-like RHG homologs in *A. gambiae* and other mosquito species (Figure 1 and Supplementary data file S1). Moreover, while no *mx*-like homologs were detectable outside the dipteran suborder Culicomorpha, two additional *mx*-like paralogs were found in members of the mosquito subfamilies Culicinae (*Aedes aegypti*, *Culex pipiens*, *Tripteroides aranoides*) and Toxorhynchitinae (*Toxorhynchites* spec.) (Figure 2). Combined, these findings uncovered an expansion of the derived *mx*-type RHG subfamily in mosquitoes, paralleling that of *rpr*, *grim*, and *skl* in the higher Diptera. Unlike in the latter case, however, the C-termini of the mosquito *mx* paralogs were characterized by a high degree of overall sequence conservation with tyrosine (Y) as the C-terminal residue (Figure 2 and Supplementary data file S2).

### 2.5. Gene Structure Conservation

**Figure 3:**
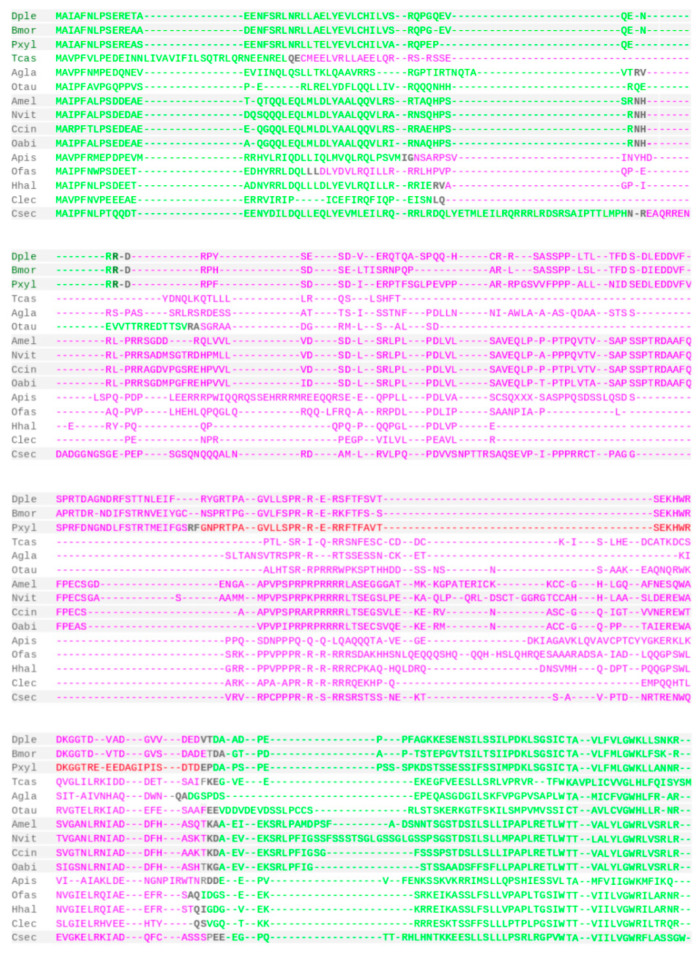
Gene structure conservation. Multiple sequence alignment of representative, newly identified RHG homologs. Exon borders indicated by black bold font. Sequence from different exons sequentially colorized green and purple. Additional exon in *P. xylostella* (Pxyl) is highlighted in red. Light grey background shades indicate different insect orders. See Supplementary data file S1 for species name abbreviations.

The open reading frames (ORFs) of *Drosophila grim*, *rpr*, and *skl* are localized on single exons, while the ORF of *hid* spreads out over four exons [8], an organization that is conserved in the *hid* ortholog of the scuttle fly *Megaselia scalaris* [20]. To probe for possibly conserved gene structures in the newly identified RHG homologs, I investigated the exon–intron organization of 15 newly identified homologs based on transcript expression (RNAseq) supported gene models in the gene database of NCBI (Figure 3). RHG homolog selection was guided by covering maximal phylogenetic depth for each order and included experimental model systems such as the silkworm moth *B. mori* [35], the red flour beetle *T. castaneum* [36], the jewel wasp *Nasonia vitripennis* [37], and the milkweed bug *Oncopeltus fasciatus* [38].

In the great majority of cases, ORFs were spread out over three exons (Figure 3). Splicing site positions, however, were only conserved in a few cases within orders, most obviously in the Lepidoptera and Hymenoptera. In the former, the homolog of the oldest clade sampled, i.e., the Yponomeutoidea, represented by the diamondback moth *Plutella xylostella*, was characterized by the acquisition of an exceptional fourth ORF encoding exon (Figure 3).

In general, the 5′- and 3′-terminal ORF segments were encoded on smaller exon contributions than the intermittent regions, which also differed by a higher level of sequence divergence. Moreover, the ORF position of the splice site linking the intermittent region with the 3′-terminal ORF segment appeared generally more strongly conserved than the positions of other splice sites. Overall, the large sample of RHG homologs was characterized by a deeply conserved gene organization that resembled that of *Drosophila hid* most closely [8].

### 2.6. A Conserved RHG Isoform in the Lepidoptera

**Figure 4:**
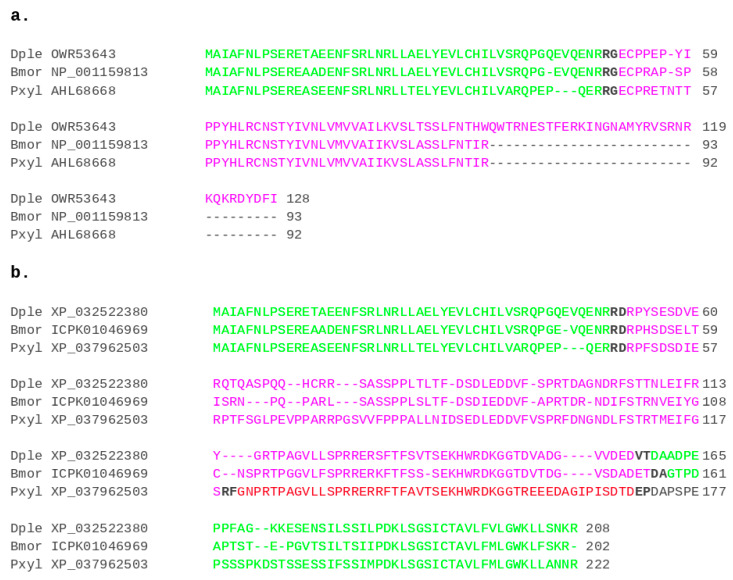
RHG protein products of conserved splice isoforms in the Lepidoptera. (**a**) Multiple sequence alignment of the RHG S-isoforms of *D. plexippus* (Dple), *B. mori* (Bmor), and *P. xylostella* (Pxyl). (**b**) Multiple sequence alignment of the L-isoform protein sequences for the same species. Exon boundary highlighting and protein sequence color coding as in Figure 2.

In many Lepidoptera, initial BLASTp searches recovered two types of RHG orthologs per species. In these cases, the two apparent homologs were sequence identical in the N-terminal region but diverged C-terminally. This preliminary evidence of differential splice isoforms was confirmed by the gene structure analyses. In the genome draft of the monarch butterfly, *Danaus plexippus*, for instance, one isoform (OWR53643.1) was identified among the curated protein sequence predictions [39] and a second (XP_032522380) among the automatic protein sequence predictions in the genome draft assembly Dplex_v4 (GenBank assembly accession: GCA_009731565.1). The same organization was eventually found for all sampled lepidopteran homologs, with one isoform being the product of run-off translation from the first exon. As the resulting predicted proteins were on average 80 amino acids shorter than those of the second isoform resulting from the 2–3 exons spanning ORFs, it seemed appropriate to name the two isoforms short (S) and long (L) RHG isoforms, respectively (Figure 4). The presence of both isoforms in the diamondback moth *P. xylostella*, i.e., the representative of the Yponomeutoidea, implied at least 140 million years of evolutionary conservation in the Lepidoptera [40].

### 2.7. Exceptional RHG Sequence Divergence in Aphids

**Figure 5:**
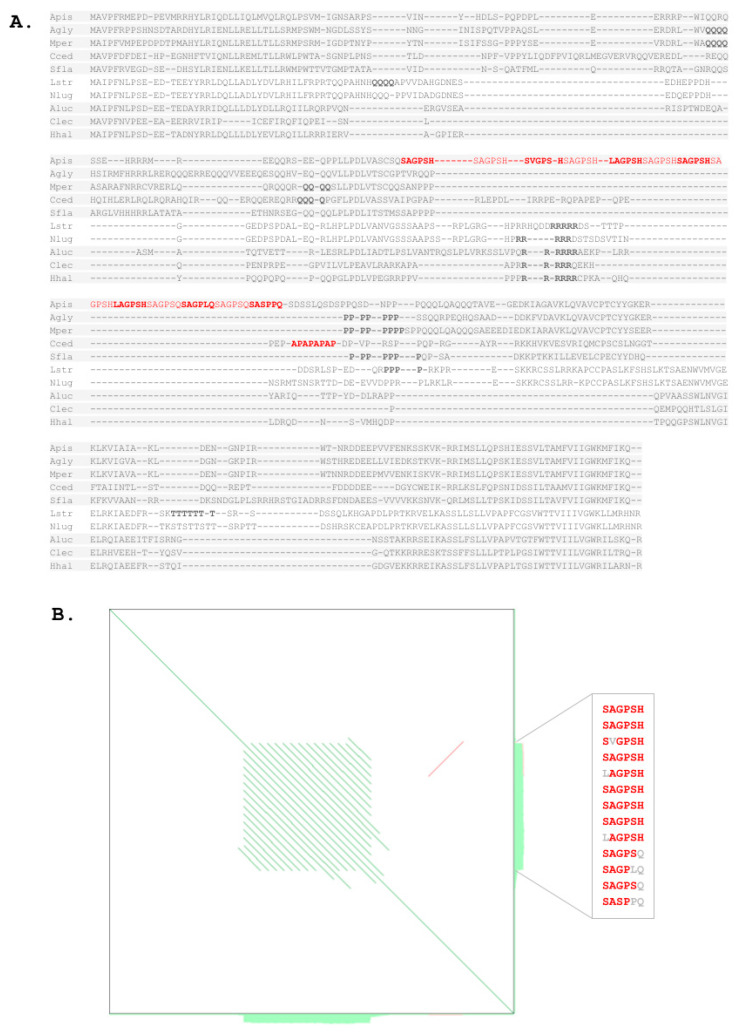
Protein sequence divergence in aphid RHG homologs. (**A**) Multiple sequence alignment of hemipteran RHG homolog protein sequences. Background shade visualizes clade composition. Top 5 species represent members of the family Aphididae (Apis = *Aphis pisum*, Agly = *Aphis glycines*, Mper = *Myzus persicae*, Cced = *Cinara cedri*, Sfla = *Sipha flava*). Species 6 and 7 from the top are planthoppers (Auchenorrhyncha: Lstr = *Laodelphax striatellus*, Nlug = *Nilaparvata lugens*). Bottom 3 species represent the suborder Heteroptera (Aluc = *Apolygus lucorum*, Clec = *Cimex lectularius*, Hhal = *Halyomorpha halys*). Single amino acid repeat strings longer than 3 residues highlighted in bold font. 13-mer repeat of the hexapeptide “(S/H)(A/V)GP(S/L/P)(H/Q)” in the pea aphid and strong of the residue duplet AP in *Cinara cedri* highlighted in red font. (**B**) Sequence similarity dot blot generated with YASS [44] of the *A. pisum* RHG coding region DNA sequence XM_001950167.5 visualizing internal repetitiveness of the 13-mer “(S/H)(A/V)GP(S/L/P)(H/Q)” repeat at the nucleotide sequence level. Green shading along blot edges indicates significantly repetitive sequence regions. Box to the right shows alignment of the 13 repeats stacked top to bottom in N- to C-terminal direction with main consensus residues highlighted by bold red font and variant residues indicated by grey font.

Past efforts failed to identify RHG homologs in the pea aphid *A. pisum*, an important pest species and genome evolution model [19,41,42]. Using the C-terminal RHG homolog region of the brown planthopper Nilaparvata lugens as query in a PSI-BLAST search against the nr database for the taxonomic range of aphid species (Aphidoidea) yielded a single hit in the yellow sugarcane aphid, Sipha flava, with an e value of 0.009. Subsequent searches with the *S. flava* RHG homolog uncovered single copy hits in nine additional aphid species including *A. pisum* (Figure 1 and Figure 5A, and Supplementary data file 1). Most of the aphid homologs were characterized by a number of glutamine (Q) and proline (P) repeat strings in the middle region of the protein, some of which were of variable lengths even between closely related species. Similar repetitive sequence elements were also found in other hemipteran RHG homologs (Figure 5A). The protein sequence of *A. pisum*, however, stood out by a unique 13 repeat units long string of the sextamer “(S/H)(A/V)GP(S/L/P)(H/Q)” with six perfect copies of “SAGPSH” (Figure 5A,B). Expression of this simple sequence region was supported by RNAseq data mapped against the gene *A. pisum* RHG gene model in the NCBI gene database (not shown). Similarity blotting of the *A. pisum* RHG coding sequence confirmed corresponding repetitiveness as the nucleotide level, which is typical for slippage extended simple sequence repeats (Figure 5B) [43].

A second unusual characteristic of the aphid RHG homologs was their consistent deployment of glutamine (Q) as the N-terminal residue in place of the deeply conserved ancestral arginine (R) residue in the Hemiptera and other insect orders (Figure 1 and Figure 5A). Combined, the stronger departure of aphid RHG homologs from some of the broadly conserved RHG sequence characteristics provided an explanation for their lower detectability with query sequences from distantly related species.

## 3. Discussion

The expanded panel of insect RHG homologs clarifies a number of previously elusive aspects of this critical cell death gene family. Most importantly, perhaps, and consistent with previous speculations [20], *hid* is now clearly established as the most ancestrally organized member of the four *Drosophila* RHG paralogs via outgroup comparison. Further significant, the protein product of *hid*, in contrast to *rpr*, *skl*, and *grim*, is localized to mitochondria due to its hydrophobic C-terminus (392–409), which has therefore been defined as the mitochondria-targeting sequence (MTS) domain [45]. Thus, while the role of mitochondria in *Drosophila* cell death is still not clearly defined, the conservation of N-terminal residues, i.e., a *hid*-like MTS domain, in ancestral RHG homologs across winged insects constitutes compelling evidence that mitochondrial localization might be a critical aspect of insect RHG protein function. The possibility that the *hid* MTS domain promotes IAP degradation by virtue of mitochondrial colocalization, therefore, continues to be an attractive model [20,46]. This is further supported by the fact that both IMB and MTS are essential for *hid*’s cell death-inducing capacity [20]. Interestingly, also, the lepidopteran S-isoform is mitochondrially localized, based on immunohistochemical detection in the armyworm moth *S. frugiperda* [26]. At first glance, this suggests a higher level of functional conservation between the derived S-isoforms and the ancestrally organized L-isoforms in the Lepidoptera compared to that between *hid* vs. *grim*, *rpr*, and *skl* in *Drosophila*.

The updated insect RHG homolog database further reveals that *rpr*, *skl*, and *grim* are not the only examples of RHG gene family expansions resulting in paralogs with simpler gene organization, i.e., a lower number of coding exons, and substantially shorter protein sequences. This is also true for the *mx* paralogs in mosquitoes and the derived S-isoforms of the lepidopteran RHG genes. The discovery of the latter further suggests that the dipteran RHG gene family expanded via the selective duplication of the first ORF sequence containing exon, which encodes the short, but cell death induction sufficient IBM. This duplication conduciveness likely explains the spawning of RHG paralogs and isoforms in mosquitoes and Lepidoptera, respectively [20].

The existence of multiple *mx* homologs in mosquitoes had been noted earlier [47]. Tissue- and, ideally, cell-specific expression studies will reveal whether and how these duplications translated into functional diversification compared to the ancestrally *hid*-like homologs of mosquitoes. While these efforts may reveal connections to the exceptional pathogen load of mosquito vector pest species, it is also possible that they represent functionally neutral outcomes of gene duplication in line with the “duplication–degeneration–complementation” trajectory [48,49]. This, in fact, could apply to *hid*, *rpr*, *grim,* and *skl*, given their largely non-overlapping expression patterns based on modENCODE data [50].

The first RHG homologs identified outside dipterans via a bioinformatic search in a new genome sequence was *Ibm1* of *B. mori* [24], which is now identified as the derived S-isoform of the *B. mori* RHG homolog locus. Paralleling the situation in mosquitoes, it is the ancestrally organized L-RHG isoform that now awaits functional study [24,26]. Future analyses of both lepidopteran isoforms have the potential to inform about the subfunctionalization trajectories of newly emerging RHG homologs. In this case, the existence of post-transcriptional mechanisms can be envisioned to confer cell- or tissue-specific functions.

It has been over 10 years since the last RHG homolog was detected in a new insect order. This hiatus is, of course, in part explained by the well recognized challenges of finding RHG homologs, i.e., their short sequence lengths, relatively unconstrained evolution, and low number of constrained residues. However, the updated RHG compilation also reveals a role of historical contingencies. The previously identified homologs in mosquitoes and lepidopterans both represent derived homologs or isoforms that lack the conserved C-terminus. This may, in part, explain subsequent failures to identify RHG homologsin Hemipterans [19]. The latter case, however, is also an example of yet another likely impeding coincidence. Some ancestrally organized RHG homologs have exceptionally diverged even in the N- and C-terminal regions, thus reducing their detectability. This is true for aphids, including *A. pisum*, arguably the genomically best documented representative of its clade [41] and the RHG homologs of darkling beetles, which includes *T. castaneum*. It is notable that the RHG homologs of both *A. pisum* and *T. castaneum* were only detected after more closely related homolog sequences were at hand as queries, a strategy that may be referred to as “taxon hopping”. Future applications of this approach will benefit from computational automatization and refinements that optimize sensitivity and specificity.

Varied BLAST searches in genome and transcriptome databases of older insect clades, i.e., Paleoptera and apterygote Hexapoda, as well as crustaceans and invertebrates in general, did not uncover further RHG homologs at this point. Given the success of the “taxon hopping” strategy in identifying new homologs, it seems reasonable to assume that the RHG gene family is restricted to neopteran insects. Thus, besides identifying new powerful insect model systems for the study of RHG function, the expanded compilation of RHG homologs suggests a new hypothetical time point of RHG family origination at the base of the Neoptera and predicts the existence of different IAP inhibiting regulators in other clades.

## 4. Materials and Methods

### 4.1. Homolog Searches

Using the BLAST search interface of the National Center for Biotechnology Information (NCBI), homolog searches were conducted with BLASTp, tBLASTn, or Position-Specific Iterated BLAST (PSI-BLAST) in the non-redundant (nr) protein sequence, Transcriptome Shotgun Assembly (TSA), and Whole Genome Shotgun contig (WGS) sequence databases [51,52,53]. Most searches were performed at default settings. In rare cases, searches were repeated with setting word size to 3 and expected threshold to 0.5.

### 4.2. Multiple Sequence Alignments

Multiple protein sequence alignments were generated using Clustal Omega, webPRANK, and T-Coffee all at default settings [54,55,56].

### 4.3. Gene Structure Analyses

Gene structures were analyzed in current assemblies available in the NCBI Genome Data Viewer [51].

## Figures and Tables

**Table 1:** Clade-specific diversification rates of RHG homologs. The dipteran sample included *D. melanogaster hid* but no homologs of *skl*, *grim*, or *rpr*. See Supplementary data files S3–S9 for corresponding MSAs. Divergence times based on [27].

	Average Lengths	% Conserved Sites	Divergence Times	% Divergence/Million Years
Diptera (*n* = 13)	302.0	17.2%	200	0.41%
Lepidoptera (*n* = 13)	210.1	54.7%	120	0.38%
Coleoptera: Polyphaga (*n* = 24)	189.3	4.2%	250	0.38%
Hymenoptera (*n* = 19)	244.9	54.3%	250	0.18%
Hemiptera wo Aphidoidea (*n* = 8)	225.5	24.4%	260	0.29%
Aphidoidea (*n* = 10)	252.8	25.3%	25	2.99%
Dictyoptera (*n* = 3)	253.3	56.1%	175	0.25%

## Data Availability

The data presented in this study are available in supplementary material.

## Supplementary Materials

The following are available online at https://www.mdpi.com/article/10.3390/insects12110957/s1. Supplementary data file S1: Protein sequence IDs and species information of RHG homolog compilation. Supplementary data file S2: MSA of culicomorph RHG protein sequences. Top five sequences: *mx* homologs from all investigated mosquito species. Red and green font: Additional *mx* homologs in species from the subfamily Culicinae. *Hid*-like homologs from mosquito and other dipteran species. See Supplementary data file S1 for species abbreviations. Supplementary data file S3: MSA of dipteran RHG protein sequences. See Supplementary data file S1 for species abbreviations. Supplementary data file S4: MSA of lepidopteran RHG protein sequences. See Supplementary data file S1 for species abbreviations. Supplementary data file S5: MSA of coleopteran RHG protein sequences. See Supplementary data file S1 for species abbreviations. Supplementary data file S6: MSA of hymenopteran RHG protein sequences. See Supplementary data file S1 for species abbreviations. Supplementary data file S7: MSA of hemipteran RHG protein sequences, Aphidoidea excluded. See Supplementary data file S1 for species abbreviations. Supplementary data file S8: MSA of Aphidoidea RHG protein sequences. See Supplementary data file S1 for species abbreviations. Supplementary data file S9: MSA of dictyopteran RHG protein sequences. See Supplementary data file S1 for species abbreviations.
