# Supplementary material for: Deep Conservation of Hid-Like RHG Gene Family Homologs in Winged Insects Revealed by “Taxon Hopping” BLAST
Source: Insects. 2021 Oct 21;12(11):957. doi: 10.3390/insects12110957 (PMC8620624; doi:10.3390/insects12110957)
Supplement: Supplementary file 1 [file insects-12-00957-s001.zip › Supplementary data file S2_ MSA of culicomorph RHG protein sequences.pdf]

```
Aaeg_mx_ABD47742          MAIAFYIP---------------------AIDDEIEK---------------Q-------
Aalb_mx_AAW72439          MAIAFYIP---------------------AIDDEIEK---------------R-------
Cpip_mx_XP_039452301      MAIAFYIP---------------------AIDDEVER---------------Q-------
Agam_mx_ABD47743          MAIAFYIP---------------------SLDDEAEN---------------L-------
Aste_mx_XP_035892327      MAIAFYIP---------------------SLDDAEEN---------------Q-------
Aaeg_GFNA01179070         MAVAFYIP---------------------SVDEEEPV---------------K-------
Aalb_GCLM01039770         MAVAFYIP---------------------SVDEDEPV---------------K-------
Cpip_XP_039438427         MAVAFNVP---------------------SVHEDDEG---------------Q-------
Aaeg_XP_021695521         MAIAFY-----------------------CED-EDPKVMF-GGSYGYHQQSYQ-------
Aalb_XP_029730094         MAIAFY-----------------------CED-EDPKMMF-SGGYHHHHQSYQWQQQPQQ
Cpip_XP_039452297         MAIAFY-----------------------CEEEEDPKGYYDSYGYGYQLQSQQ-------
Asus_D_AEF30451           MAVVFYMPE-------------------AGADD---------------------------
Bdor_D_AHX71830           MAVVFYMPE-------------------GGGDD---------------------------
Lser_D_AKA64736           MAVPFYLPE-------------------GGADD---------------------------
Bodo_D_KAG4071353         MAVPFFVPD-------------------G--DD---------------------------
Aste_XP_035899618         MAKPFMLPE-------------------SGPGG---------------------------
Agam_GIBN01014598         MAKPFSLPE-------------------PGPGG---------------------------
Aalb_D_XP_029727134       MAIPFLQPEEDESARHHGANGPNTASAHAGVGA---------------------------
Aaeg_XP_021705280         MAIPFLQPEEDESARH--GNGQ---LVHAGAGA---------------------------
Cpip_XP_039434833         MAIPFLQPEDDDAAR----------LQHA-------------------------------

Aaeg_mx_ABD47742          YQ--LQMMQQQQ----------QILMQ------Q--Q-QN----------------QNQI
Aalb_mx_AAW72439          YQ--LQ----QQ----------LILAQR-----Q--Q-QN----------------QQQF
Cpip_mx_XP_039452301      HQ--LQ----LQ----------Q---Q------Y--Q-QY----------------QHQ-
Agam_mx_ABD47743          RL--LQ----QQ----------QMVLQ-RQYLLQ--Q-QYHLQAQLNLVHQQQLALEQQ-
Aste_mx_XP_035892327      RL--LE----QQ----------QIVLQ-RQYLLQ--Q-QYHLQAQMNQLRQQQLALEQQ-
Aaeg_GFNA01179070         PN--HP----QQ-------------VG------Q--Q-QH----------------QQQ-
Aalb_GCLM01039770         PNSPHP----QQ-------------VG------G--G-QH----------------QQA-
Cpip_XP_039438427         WK--PA----NQ-------------LG------QPNQ-QP----------------QHQ-
Aaeg_XP_021695521         SQ--HS----VA-------------VG------Q--QHAG----------------TGE-
Aalb_XP_029730094         TQ--HA----VA-------------VG------Q--QHAG----------------SGS-
Cpip_XP_039452297         SQ--HA----ATAPPATGSQQP---VA------Q--Q-AA----------------SGD-
Asus_D_AEF30451           ------------------------------------------------------------
Bdor_D_AHX71830           ------------------------------------------------------------
Lser_D_AKA64736           ------------------------------------------------------------
Bodo_D_KAG4071353         ------------------------------------------------------------
Aste_XP_035899618         ------------------------------------------------------------
Agam_GIBN01014598         ------------------------------------------------------------
Aalb_D_XP_029727134       ------------------------------------------------------------
Aaeg_XP_021705280         ------------------------------------------------------------
Cpip_XP_039434833         ------------------------------------------------------------
Aaeg_mx_ABD47742          SSPP--------------------------------------------------------
Aalb_mx_AAW72439          SSPP--------------------------------------------------------
```

```
Cpip_mx_XP_039452301     -SPP--------------------------------------------------------
Agam_mx_ABD47743         -SAA-ISTN----------------TAAP--------G--TA---GP-------------
Aste_mx_XP_035892327     -SAA-ISTT----------------AGTS--------GASST---GS-------------
Aaeg_GFNA01179070        -S----------------------------------------------------------
Aalb_GCLM01039770        -S----------------------------------------------------------
Cpip_XP_039438427        -H----------------------------------------------------------
Aaeg_XP_021695521        -G----------------------------------------------------------
Aalb_XP_029730094        -G----------------------------------------------------------
Cpip_XP_039452297        -D----------------------------------------------------------
Asus_D_AEF30451          -SASSSSGS----------------HGSG--------GSGSA---AS-----S-------
Bdor_D_AHX71830          -SASSSSGG----------------HGSA--------GSGSA---AS-----S-------
Lser_D_AKA64736          -TVS-SGGT----------------TSSD--------GASSA---AS-----S-------
Bodo_D_KAG4071353        -QRS-NG---------------------------------------------S-------
Aste_XP_035899618        -GSS-SSGA-GPP-SNAPSNTNTVAGNTAPSTPA-PIGTGSA---ASN--GPSGASGHVS
Agam_GIBN01014598        -GSS-SSGA-GPPANNVSSNSNAAAGGTAPSTPALQNGSGSG---ASHPANPAGPLGHPP
Aalb_D_XP_029727134      -GAG-SSGTG---------------SASASGSGA-VVVGGAG---SS-----GGAVM---
Aaeg_XP_021705280        -GAG-SSAA----------------SGSAGSSSG-VVGAGSGIAASG-----GGAVM---
Cpip_XP_039434833        --------A----------------TQSGPPTLG-PVSPGPG---SS-----GGPLLHPP

Aaeg_mx_ABD47742         ------------------------------------------------------------
Aalb_mx_AAW72439         ------------------------------------------------------------
Cpip_mx_XP_039452301     ------------------------------------------------------------
Agam_mx_ABD47743         ------------------------------------------------------------
Aste_mx_XP_035892327     ------------------------------------------------------------
Aaeg_GFNA01179070        ------------------------------------------------------------
Aalb_GCLM01039770        ------------------------------------------------------------
Cpip_XP_039438427        ------------------------------------------------------------
Aaeg_XP_021695521        ------------------------------------------------------------
Aalb_XP_029730094        ------------------------------------------------------------
Cpip_XP_039452297        ------------------------------------------------------------
Asus_D_AEF30451          ------------------------------------------------------------
Bdor_D_AHX71830          ------------------------------------------------------------
Lser_D_AKA64736          ------------------------------------------------------------
Bodo_D_KAG4071353        ------------------------------------------------------------
Aste_XP_035899618        SAPI-----SVTGANSSQQYHLQQLHHHHQQLQQHYQSSYQGSHPALMTAPGPGSVP--V
Agam_GIBN01014598        SAPI-----PMPVPTSPQQYHLQQLHHHHQQLQQHYQ-AYQGPHGTLMAAPPGSSVA--V
Aalb_D_XP_029727134      ---LAGATGPMPVPNSP--------------------------------------AAPII
Aaeg_XP_021705280        ---MVGATGPLPIPNSP--------------------------------------AP--I
Cpip_XP_039434833        PSPLV--SGSIPIPGSP--------------------------------------AP--I
Aaeg_mx_ABD47742         -----------------------P------------------------------------
Aalb_mx_AAW72439         -----------------------P------------------------------------
Cpip_mx_XP_039452301     -----------------------P------------------------------------
Agam_mx_ABD47743         ----------------NAATVTAA------------------------------------
```

```
Aste_mx_XP_035892327      ----------------TASGVAVP------------------------------------
Aaeg_GFNA01179070         ------------------------------------------------------------
Aalb_GCLM01039770         ------------------------------------------------------------
Cpip_XP_039438427         ------------------------------------------------------------
Aaeg_XP_021695521         ------------------------------------------------------------
Aalb_XP_029730094         ------------------------------------------------------------
Cpip_XP_039452297         ------------------------------------------------------------
Asus_D_AEF30451           --TS-------QSPNTTTSATQTPMQSP---LMASP-HIVMALCEAVYQ---SLN-----
Bdor_D_AHX71830           --TS-------QSPNTTTSATQTPMQSP---L--PP-HFMMAF----YQ---YMN-----
Lser_D_AKA64736           --LQ-------SSPNTTSSATQTPMQSP---L--LPGEVIMAI----YELIRYNS-----
Bodo_D_KAG4071353         --TP-------QSQSTISSATQTPLQSP---V--NDEHLVQAF----YE---ALSLYSAN
Aste_XP_035899618         -VDPHLLLPMTPPASGFATAIQTPQMAP---V--N-EQLVQAL----YE---AIYRCSWG
Agam_GIBN01014598         PVDPHLMLPMTPPPSGFATTVQTPQIPP---V--N-EQLVQAL----YE---AIYRCSLG
Aalb_D_XP_029727134       -------------SSGTSTAVQTPLIGP---M--NSDQLVQLF----YE---ALYRYSRG
Aaeg_XP_021705280         -------------SSGTSTAVQTPLIGP---M--NSDQLVQLF----YE---ALYRYSRG
Cpip_XP_039434833         GVPP-------PPPSGTSTAVQTPLLGPAGLM--HGDQLVQLF----YE---ALYRYSRG

Aaeg_mx_ABD47742          -S-PTSPTESIPP-----TPPLTPT---MT------------------------------
Aalb_mx_AAW72439          -S-PT---ESIPP-----TPPLTPT---MT------------------------------
Cpip_mx_XP_039452301      -T-PTTPTESIPP-----TPPHTPT---MT------------------------------
Agam_mx_ABD47743          -T-PQPPAASMPPSTT--TNTQIPS---MV-------SA---------------------
Aste_mx_XP_035892327      -TVPTLTTPSAPPTST--TPQQPPS---TS----IPPNA---------------------
Aaeg_GFNA01179070         -----------------------PN---QT-------TS---------------------
Aalb_GCLM01039770         ------------------------N---QT-------TG---------------------
Cpip_XP_039438427         -----------------------PH---HHHHVG---AS---------------------
Aaeg_XP_021695521         -----------------------TT---DE-------NN---------------------
Aalb_XP_029730094         -----------------------GT-VVDE-------NN---------------------
Cpip_XP_039452297         -----------------------EN---NN-------NS---------------------
Asus_D_AEF30451           ----NTPAFQYPPPASPCCHVHSPSP--YT-------GSG-GGG----------------
Bdor_D_AHX71830           ----AT-AFQYPPPASPCCQVHSPSP--YT-------GGG-GGGGGGGAGVGNG------
Lser_D_AKA64736           ----GAPVFQYPTPPSPSCSIHGPS---YT-------G-----G----------------
Bodo_D_KAG4071353         LL-QATPSCLSPPSSPSCCHRHN-N---YR-------G----------------------
Aste_XP_035899618         DG-PPTPLSQSPPSAS--CSPFTTG---AY-------Y---GSG----------------
Agam_GIBN01014598         DV-PTTPLSQSPPSAS--CSPFANG---NF-------F---GSG----------------
Aalb_D_XP_029727134       DT-PSTPISQSPQSSS--CSPFTTG---YY-------NSG-GGG----------SAGYAN
Aaeg_XP_021705280         DT-PSTPISQSPQSSS--CSPFTTG---YY-------NSGGGGG----------SAGFVN
Cpip_XP_039434833         DT-PSTPISQSPQSSS--CSPFTTG---YY-------NTP-PGG----------------
Aaeg_mx_ABD47742          -----------------------------------------------QVSL------HH-
Aalb_mx_AAW72439          -----------------------------------------------QVSI------HH-
Cpip_mx_XP_039452301      -----------------------------------------------QVSI------HH-
Agam_mx_ABD47743          -------------AGS------T-----Q------------------QQHQ------QH-
Aste_mx_XP_035892327      -------------ASS------SYYTAPA------------------PQHA------QH-
Aaeg_GFNA01179070         -------------SDL------S-----Q------------------QVCAEAEAVAEL-
```

```
Aalb_GCLM01039770          -------------SNV------S-----Q------------------QVCA-AEAVAEY-
Cpip_XP_039438427          -------------TNV------S-----Q------------------QVRL-S---AEN-
Aaeg_XP_021695521          -------------NNC--ALHGS-----NNGGGGGGDGESGVSGGEQQSSA-T---MVNS
Aalb_XP_029730094          -------------NNC--ALHG---------GGGGESGENGVE--QQQSSA-T---VAHS
Cpip_XP_039452297          -------------NNC--ALHGS-----S------------------PLTA-D---VVN-
Asus_D_AEF30451            -----EVFFPPSRST-------P-----R------------------TPRT-S---VSF-
Bdor_D_AHX71830            -----EVFFPLSLST-------P-----R------------------TPRT-S---VSY-
Lser_D_AKA64736            -----EVFFPRSRST-------P-----R------------------IPRT-S---VSF-
Bodo_D_KAG4071353          -----DVFFPQTHSS-------P-----R------------------TPSA-S-------
Aste_XP_035899618          -----QVFFPRTPANT-----------------------------------------AN-
Agam_GIBN01014598          -----QVFFPRTPANT-----------------------------------------AN-
Aalb_D_XP_029727134        ATTTGQVFFPQTPNGVFR----P---------------------------------GGG-
Aaeg_XP_021705280          TGTTSQVFFPQTPNGVYR----P---------------------------------S---
Cpip_XP_039434833          -----QVFFPQTPSGVLP----P-----R------------------TPNA-T---GGG-

Aaeg_mx_ABD47742           -N----RYH----LVQ--A-FHE------ILFR--HAQ----------------------
Aalb_mx_AAW72439           -N----RYH----LVQ--A-FHE------ILFR--HAQ----------------------
Cpip_mx_XP_039452301       -N----RYH----LVQ--A-FHE------ILFR--HAQ----------------------
Agam_mx_ABD47743           -HHQHQRFQ----VVR--AVLHD------ILFR--HLL----------------------
Aste_mx_XP_035892327       -H----RFW----MLR--GILYN------ILFR--HLL----------------------
Aaeg_GFNA01179070          -Q----R-H----HLM--VVFHE------ILVA--YAR----------------------
Aalb_GCLM01039770          -Q----R-Q----HLI--AVFHE------ILVA--YAR----------------------
Cpip_XP_039438427          -G----Q-H----RLV--AAFHQ------ILAA--YAR----------------------
Aaeg_XP_021695521          SN----R-Q----LMR---VLHQ------VLYR--YTM----------------------
Aalb_XP_029730094          SN----R-Q----LMR---VLHQ------VLYR--YTM----------------------
Cpip_XP_039452297          -N----R-Q----LMR--VVLHQ------VLYR--CTM----------------------
Asus_D_AEF30451            -A----A-G----EEN--TFFRQ---------H--NISGGYSSTQHLQQPPQSAPPMPPS
Bdor_D_AHX71830            -A----A-G----EEN--TFFRH---------H--NISGGCSNASHL-QPPQSAPPMPSS
Lser_D_AKA64736            -A----P-G----DEV--NFFHQ---------SAPAASSGNAQTQTT-QAPQSAPPVPNS
Bodo_D_KAG4071353          ------------------------------------------------------------
Aste_XP_035899618          -G----R-N----GAR--VFFHQT--PGQ---Q--YPQV----PQSQ-PTPGSA----NS
Agam_GIBN01014598          -G----R-N----GAR--VFFQQTPNPGP---Q--YTPVGNSQSQQQ-PPPGSA----NS
Aalb_D_XP_029727134        -G----G-GIGSYGATTGVFFGQP--P-------------------T-PSPSGS----SS
Aaeg_XP_021705280          --------GVGSFG-TTGVFFGQP--P-------------------T-PSPSGS----SS
Cpip_XP_039434833          -G----R-G----GAR--IFFGQ---P-------------------T-PSPSGS----SS
Aaeg_mx_ABD47742           -------E--N--N---------P------------------------------------
Aalb_mx_AAW72439           -------E--N--N---------P------------------------------------
Cpip_mx_XP_039452301       -------E--N--S---------P------------------------------------
Agam_mx_ABD47743           -------D--T--S---------A------------------------------------
Aste_mx_XP_035892327       -------D--N--D---------P------------------------------------
Aaeg_GFNA01179070          -------E--AALAQHSPNPKALP------------------------------------
Aalb_GCLM01039770          -------E--AALAQHSPNPKALPP-----------------------------------
Cpip_XP_039438427          -------H--AILAQEQQQTTTTT------------------------------------
```

```
Aaeg_XP_021695521        -------E--GVLS---------P------------------------------------
Aalb_XP_029730094        -------E--GVLS---------P------------------------------------
Cpip_XP_039452297        -------E--GALS---------P------------------------------------
Asus_D_AEF30451          SHTSAHSQ--T--T---------P-Q-----Q-Y-QQYSYPHYQYTPPPTPLTANASTCT
Bdor_D_AHX71830          SHNGAHSQ--P--A---------P-Q-----QQY-QQYTYPYYQYTPPPTPLTANASTCT
Lser_D_AKA64736          HHHNQHQLYGH--H---------P-H-----FAY-QHSGPNYYQYTPPPTP---NA----
Bodo_D_KAG4071353        ------------------------------------------------------------
Aste_XP_035899618        HSSSSGVS--S--S---------L-D-----TPS-CSSGTPHYHH---------------
Agam_GIBN01014598        HSSSSGVS--S--S---------V-D-----TPS-CSSGTPHYHQ---------------
Aalb_D_XP_029727134      H-S-QHSA--N--S---------P-D-----TAA-CSSAPPIIHQFPSSYQ---LP----
Aaeg_XP_021705280        HSS-QHSA--N--S---------P-E-----TPA-CSSAPPIFHQFPSSYQ---LP----
Cpip_XP_039434833        YSSSQHSA--N--S---------P-DASHTTTPASCSSAAPIFHQFPSSFS---GP----

Aaeg_mx_ABD47742         -----------------------TR-------------------------------SR--
Aalb_mx_AAW72439         -----------------------TR-------------------------------SR--
Cpip_mx_XP_039452301     -----------------------TR-------------------------------TR--
Agam_mx_ABD47743         -----------------------SAPKKKKRKPKPPRIYNNNYYYNYYCRNISHHFLR--
Aste_mx_XP_035892327     -----------------------AC-------------------------------IR--
Aaeg_GFNA01179070        -----------------------TT-------------------------------EP--
Aalb_GCLM01039770        -----------------------TT-------------------------------EP--
Cpip_XP_039438427        -----------------------TS-------------------------------VPPP
Aaeg_XP_021695521        -----------------------AQ-------------------------------TS--
Aalb_XP_029730094        -----------------------AQ-------------------------------TS--
Cpip_XP_039452297        -----------------------AH-------------------------------TS--
Asus_D_AEF30451          GNVFGSTEATTHQRTCV--ASTSTS-------------------------------TQ--
Bdor_D_AHX71830          GNIFGATEATTHQRGCVATASTSTS-------------------------------TT--
Lser_D_AKA64736          -----------------ATATTSTG-------------------------------TS--
Bodo_D_KAG4071353        ------------------------D-------------------------------TS--
Aste_XP_035899618        ------------------------------------------------------------
Agam_GIBN01014598        ------------------------------------------------------------
Aalb_D_XP_029727134      -----------------STSNASTA-------------------------------TN--
Aaeg_XP_021705280        -----------------STANASTT-------------------------------TN--
Cpip_XP_039434833        ------------------TADGGST-------------------------------TS--
Aaeg_mx_ABD47742         -CLLCNK------------LYYLLRKVY--------------------------------
Aalb_mx_AAW72439         -CLLCNK------------LYYLLRKVY--------------------------------
Cpip_mx_XP_039452301     -CLLCNK------------LYYLLRKVY--------------------------------
Agam_mx_ABD47743         -CFYRHK--DDEEGGGGRLPIETPRH----------------------------------
Aste_mx_XP_035892327     -CVFCHK------------LLRMLRNIC--------------------------------
Aaeg_GFNA01179070        -CLLCKK------------LYYLFRRFY--------------------------------
Aalb_GCLM01039770        -CLLCKK------------LCYLFRRFY--------------------------------
Cpip_XP_039438427        SCVQCEK------------LHYLLRLLH--------------------------------
Aaeg_XP_021695521        -CVFCNR------------LYYLFQLLY--------------------------------
Aalb_XP_029730094        -CVFCNR------------IYYLFQLLY--------------------------------
```

```
Cpip_XP_039452297         -CVFCNK------------IYYLFQLLY--------------------------------
Asus_D_AEF30451           -SCMAKG------------HARLHRSFSDAQKRSRRTSTTNDDERECHSEHETSWDEF--
Bdor_D_AHX71830           -SCGMGG------------HSRLHRSLSDAQKRSRRTSTTNDDEREYHSEHETSWDEF--
Lser_D_AKA64736           -TSGAGGMA----------HRSLHRSLSDSARRSRLTS-TGEDEREYQSEHETSWDEF--
Bodo_D_KAG4071353         -QCW--------------------RSRANGLRR-RLHS-TGDDT---HDDAESS-DEFSM
Aste_XP_035899618         -----GQ------------LQPIQFNWHDD----------GDSS---ESEGSSS--QTTS
Agam_GIBN01014598         -----GQ------------LQPIQFNWHDD----------GDSS---ESEASTS--QATS
Aalb_D_XP_029727134       -WTH---------------VS----S--GD----------GDDS----SESDSS--QPSS
Aaeg_XP_021705280         -WSHHGG------------PS----G--HD----------GDES----SESDSS--QPSS
Cpip_XP_039434833         -PAMAGQ------------PT----SWPDD----------GSES----SESDSS--QPSS

Aaeg_mx_ABD47742          ------------------------------------------------------------
Aalb_mx_AAW72439          ------------------------------------------------------------
Cpip_mx_XP_039452301      ------------------------------------------------------------
Agam_mx_ABD47743          ------------------------------------------------------------
Aste_mx_XP_035892327      ------------------------------------------------------------
Aaeg_GFNA01179070         ------------------------------------------------------------
Aalb_GCLM01039770         ------------------------------------------------------------
Cpip_XP_039438427         ------------------------------------------------------------
Aaeg_XP_021695521         ------------------------------------------------------------
Aalb_XP_029730094         ------------------------------------------------------------
Cpip_XP_039452297         ------------------------------------------------------------
Asus_D_AEF30451           ------------------------------------------------------------
Bdor_D_AHX71830           ------------------------------------------------------------
Lser_D_AKA64736           ------------------------------------------------------------
Bodo_D_KAG4071353         AN--------------GC------------------------------------------
Aste_XP_035899618         SS--------------GCNPHCLG-TM------------------------DGGASSSSY
Agam_GIBN01014598         SS--------------GCSPHCLG--M------------------------DGSGPSSSF
Aalb_D_XP_029727134       ASDGDSITAPI----AGCSPHCLGL-LGPPH----HHLHQLLDAAAAAGSGSSTGVSSSF
Aaeg_XP_021705280         ISEGDTIAAPI----AACSPHCLGL-LPGAH----HH---LLDA------GSSTSTST--
Cpip_XP_039434833         SSE----AAPTGSSPTGCSSHCLG--LGQLHQHRLHHHQQPLDV------GGGGGPS---
Aaeg_mx_ABD47742          ------------------------------------------------------------
Aalb_mx_AAW72439          ------------------------------------------------------------
Cpip_mx_XP_039452301      ------------------------------------------------------------
Agam_mx_ABD47743          ------------------------------------------------------------
Aste_mx_XP_035892327      ------------------------------------------------------------
Aaeg_GFNA01179070         ------------------------------------------------------------
Aalb_GCLM01039770         ------------------------------------------------------------
Cpip_XP_039438427         ------------------------------------------------------------
Aaeg_XP_021695521         ------------------------------------------------------------
Aalb_XP_029730094         ------------------------------------------------------------
Cpip_XP_039452297         ------------------------------------------------------------
Asus_D_AEF30451           --------------------------------DDRYDNFTAGRERLQEFNGRIPPRKKK-
```

```
Bdor_D_AHX71830          --------------------------------DDRYDNFTAGRERLQEFNGRIPPRKKK-
Lser_D_AKA64736          --------------------------------EDRYDNFTAGRERLQEFNGRIPPRKKK-
Bodo_D_KAG4071353        NVPRQS-------------------VPVDYDLEEHY-TYLAAREHLQPFSGQVPPKKKK-
Aste_XP_035899618        SVPSSANLI----SPSSSGGTLYASVPPHLD-------YMAGREQLQEFPGKIPQRKRK-
Agam_GIBN01014598        STPSSSTVM----SPSSSGGTLYAAVPPHLD-------YMAGREQLQEFPGKIPQRKRK-
Aalb_D_XP_029727134      NNGSSSSSSSNSAGGSSAMVPMSGCIPPHLD-------YMAGREQLQEFAGKIPQRKKKS
Aaeg_XP_021705280        -IGANSSSS----GSSSTMATMSGYIPPHLD-------YMAGREQLQEFAGKIPQRKKKS
Cpip_XP_039434833        -----------------------GFIPPHLD-------FMAGREQLQEFAGKIPQRKKK-

Aaeg_mx_ABD47742         -----------------------------------------------------------
Aalb_mx_AAW72439         -----------------------------------------------------------
Cpip_mx_XP_039452301     -----------------------------------------------------------
Agam_mx_ABD47743         -----------------------------------------------------------
Aste_mx_XP_035892327     -----------------------------------------------------------
Aaeg_GFNA01179070        -----------------------------------------------------------
Aalb_GCLM01039770        -----------------------------------------------------------
Cpip_XP_039438427        -----------------------------------------------------------
Aaeg_XP_021695521        -----------------------------------------------------------
Aalb_XP_029730094        -----------------------------------------------------------
Cpip_XP_039452297        -----------------------------------------------------------
Asus_D_AEF30451          --KDVP-KS--------KSEKK----QFKAF-----IWP-TVVTVI-VVAMGCGFLVAR
Bdor_D_AHX71830          --KDVP-KS--------KYEKKVRERPFKGF-----IWP-TVVTVI-VVAIGCGFLVTR
Lser_D_AKA64736          --KDVP-KT--------KPEKK----E-QSF-----TWP-AVVTVF-VFAMGCGFLVAR
Bodo_D_KAG4071353        --RVVE-HTEADSPVATKSKSG----R-QGG-----YYA-NYLQI--------------
Aste_XP_035899618        -----S-RT---------PKPK----K-AGLLEQPLMWQ-MAVTAAALIVLGCGYLAAR
Agam_GIBN01014598        -----S-RT---------PKPK----K-PGFLEQPLMWQ-MAVTAA-LIVLGCGYLAAR
Aalb_D_XP_029727134      SS---S-KS--------SSKSK----K-SKFLEQTLVWPTLAVTAT-LIALGCGLLAAR
Aaeg_XP_021705280        SS---SSKS--------SSKSK----K-SKFLEQTLVWPTLAVTAT-LIALGCGLLAAR
Cpip_XP_039434833        -----S-KA--------GGKSK----K-AGFLEQTLVWPTLAVTAT-LIALGCGLLAAR
```
