# Supplementary material for: Deep Conservation of Hid-Like RHG Gene Family Homologs in Winged Insects Revealed by “Taxon Hopping” BLAST
Source: Insects. 2021 Oct 21;12(11):957. doi: 10.3390/insects12110957 (PMC8620624; doi:10.3390/insects12110957)
Supplement: Supplementary file 1 [file insects-12-00957-s001.zip › Supplementary data file S3_ MSA of dipteran RHG protein sequences.pdf]

```
Cpip_D_XP_039434833        MAIPFLQPEDDDAARLQHAAT---QSGPPTLG---PVSP---------------------   33
Aead_D_XP_029727134        MAIPFLQPEEDESARHHGANG---PNTASAHA---GVGAGAGSSGTGSASASGSGAVVVG   54
Bcop_D_XP_037040268        MAVPFYVPDEE--RENG-------------------------------------------   15
Bcop_D_XP_037050104        MAVPFFVPDGDDQRSNG-------------------------------------------   17
Cnas_D_XP_031624166        MAVGFFADDGDDHL----------------------------------------------   14
Dmel_hid                   MAVPFYLPEGGADDVASSSSGASGNSSPHNHPLPSSASSSVSSSGVSSASASSASSS---   57
Hill_D_XP_037904604        MAVPFYLPNQDEQGA---------------------------------------------   15
Bdor_D_AHX71830            MAVVFYMPEGGGDDSASSSSGGHGSAG---------------------------------   27
Asus_D_AEF30451            MAVVFYMPEAGADDSASSSSGSHGSGG---------------------------------   27
Ccap_D_JAB92755            MAVVFYMPEGGGDDTASSSSGGHSSTG---------------------------------   27
Msca_D_APT68153            MAVPFYMPNQEDGD----------------------------------------------   14
Lser_D_AKA64736            MAVPFYLPEGGADDTVSS-GGTTSSDG---------------------------------   26
Gfus_D_XP_037879387        MAVPFYLPEGGADDTVAS-AAGLASDG---------------------------------   26
                           **: *   :


Cpip_D_XP_039434833        GPGSSGGPLLHPPPSPLVSGSIPIPGSPAPIGVPPPPPSGTSTAVQTPLLGPAGLMHGDQ   93
Aead_D_XP_029727134        GAGSSGGAVMLA----GATGPMPVPNSPA----APIISSGTSTAVQTPLIGP---MNSDQ   103
Bcop_D_XP_037040268        --------------------------------SSSQPQSTTTSATQTPLQSPTN----EH   39
Bcop_D_XP_037050104        --------------------------------STPQSQSTISSATQTPLQSPVN---DEH   42
Cnas_D_XP_031624166        ------------------------------NSSYHTTVITTNSATQTPLQSPVS---HEY   41
Dmel_hid                   SSASSDG---------------------ASSAASQSPNTTTSSATQTPMQSPLP-T--DQ   93
Hill_D_XP_037904604        ----SGS---------------------SGSSTGSSPN-TTTSATQTPLPSPAP-VNGEQ   48
Bdor_D_AHX71830            ----SGS---------------------AASSTSQSPN-TTTSATQTPMQSPLP---PHF   58
Asus_D_AEF30451            ----SGS---------------------AASSTSQSPN-TTTSATQTPMQSPLM---ASP   58
Ccap_D_JAB92755            ----SGS---------------------AASSTSQSPN-TTTSATQTPMQSPLV---PNP   58
Msca_D_APT68153            ----------------------------GSSSSSQSPN-TSTSATQTPLQSPVT---GEY   42
Lser_D_AKA64736            ----ASS---------------------AASSLQSSPN-TTSSATQTPMQSPLL---PGE   57
Gfus_D_XP_037879387        ----TGS---------------------TSSSSQSSPN-TTSSATQTPIQSPLV---PGD   57
                                                                    .:*.***: .*
```

```
Cpip_D_XP_039434833           LVQLFYEALYRYSRGDTP-STPISQ--SPQSSSCSPFTTGYYNTPP-----------GGQ   139
Aead_D_XP_029727134           LVQLFYEALYRYSRGDTP-STPISQ--SPQSSSCSPFTTGYYNSGGGGSAGYANATTTGQ   160
Bcop_D_XP_037040268           LVQVFYEALSLYSASLVL-RSPCS-TSPPSSPSCCHPHINN---------------YRGN   82
Bcop_D_XP_037050104           LVQAFYEALSLYSANLLQ-ATPAC-LSPPSSPSCCHRH-NN---------------YRGD   84
Cnas_D_XP_031624166           LVQAFCEALHRCS----IAGSPGIQSVPP-SPSCSHP------------------NGHGE   78
Dmel_hid                      VLYALYEWVRM--YQSQQSAPQIFQYPPP-SPSCNFT--------------------GGD   130
Hill_D_XP_037904604           LVQAFMEVLYRCSSSTSNGGSPGFQFPPP-SPSCNLNSPGGF--------------YSDE   93
Bdor_D_AHX71830               MMA------FYQ-----YMNATAFQYPPPASPCCQVHSPSPYTGGGGGGGGGAGVGNGE   107
Asus_D_AEF30451               HIVMALCEAVYQS----LNNTPAFQYPPPASPCCHVHSPSPYTGS---GG-------GGE   104
Ccap_D_JAB92755               YMVALCEALLYQS----MNNPPAFQYPPPASPCCPVHSPSPFTGGSVGGA-------NGE   107
Msca_D_APT68153               LVQAICEALHRCS------------GSYPPSPSCSVHSPCYN---------------GGD   75
Lser_D_AKA64736               VIMAIYEL---IRY---NSGAPVFQYPTPPSPSCSIHGPSYT---------------GGE   96
Gfus_D_XP_037879387           LVMAFCEAFYRYSI---NSGAPVFQYPTPQSPSCSVHSPSYV---------------GGD   99
                               :                          * * .*                        .:

Cpip_D_XP_039434833           VFFPQT---PSGVLPPRTPNATGGGGRGGARIFFGQP--TPSPSGSSSYSSSQHSANSPD   194
Aead_D_XP_029727134           VFFPQT---PNGVFRPGGGGGGIGSYGATTGVFFGQPP-TPSPSGSS--SHSQHSANSPD   214
Bcop_D_XP_037040268           VFFPRP------HVAPQTPVSFG---R---------------------------------   100
Bcop_D_XP_037050104           VFFPQT------NSSPRTPSASD---T---------------------------------   102
Cnas_D_XP_031624166           VFFPRGSNSSNGSGSSSTPS----------------------------------------   98
Dmel_hid                      VFFPHGHPNPNSNPHPRTPRTSVSFSSGEEYNFFRQQQPQPHPSY----PA--PSTPQPM   184
Hill_D_XP_037904604           VFFPRN------RSTPRSPRNSFH----DEGAFFRHPIPTPSRSM---------------   128
Bdor_D_AHX71830               VFFPLS------LSTPRTPRTSVSYAAGEENTFFRHHNISGGC----------SNASHLQ   151
Asus_D_AEF30451               VFFPPS------RSTPRTPRTSVSFAAGEENTFFRQHNISGGYS---------STQHLQQ   149
Ccap_D_JAB92755               VFFPIS------RSTPRTPRTSVSFAAGEENTFFRHHNISGGGGG--GCSS--SSATHLQ   157
Msca_D_APT68153               IFFPRD------RSTPLSFSRQQ--------------Q----------------------   93
Lser_D_AKA64736               VFFPRS------RSTPRIPRTSVSFAPGDEVNFFHQSAPAASSGNAQTQT--------TQ   142
Gfus_D_XP_037879387           VFFPRS------RSTPRTPRTSVSFAAGDEVNFFHQSSPAASSSNLQPEPN--NSNSSNQ   151
                              :***
```

```
Cpip_D_XP_039434833        ASHTTTPASCSSAA--------PIFHQFPS-----------SFSGPTADGGSTTSPAMAG   235
Aead_D_XP_029727134        ------TAACSSAP--------PIIHQFPS-----------SYQLPSTSNAST-------   242
Bcop_D_XP_037040268        ------------------------------------------------------------   100
Bcop_D_XP_037050104        ------------------------------------------------------------   102
Cnas_D_XP_031624166        PPS-TP---SSN------STVIRMHDDYRS------------------------------   118
Dmel_hid                   PPQSAPPMHCSHSYPQQSAHMMPHHSA----PFGMGGTYYAGYTPPPTPNTASAGTSSSS   240
Hill_D_XP_037904604        ------------------------------------------------------------   128
Bdor_D_AHX71830            PPQSAPPMPSSSHNGAHSQPA-------PQQQYQQYTYPYYQYTPPPTPLTANASTCTGN   204
Asus_D_AEF30451            PPQSAPPMPPSSHTSAHSQTT-------PQ-QYQQYSYPHYQYTPPPTPLTANASTCTGN   201
Ccap_D_JAB92755            PPQSAPAMQQSSHNNAHSQTA-------PQ-QYQQYTYPYYQYTPPPTPLTANASTCTGG   209
Msca_D_APT68153            ------------------------------------------------------------   93
Lser_D_AKA64736            APQSAPPVPNSHH--HNQHQLYGHHPHFA---YQHSGPNYYQYTPPPTPNAATATTSTGT   197
Gfus_D_XP_037879387        PPQSAPPTVNNSSHQHHYHSPFPLHPRVAAYHHPNTASGYCQYTPPPTPTTATTST----   207

Cpip_D_XP_039434833        QPTSWP----DDGSESSESDSSQPSSSSEAAPTGSSPTGCSSHCLGLGQLHQHRLHHHQQ   291
Aead_D_XP_029727134        -ATNWTHVSSGDGDDSSESDSSQPSSASDGDSITAPIAGCSPHCLGLLGPPHHHLHQLLD   301
Bcop_D_XP_037040268        ----------------------------------------------------SHQWSTPR   108
Bcop_D_XP_037050104        ----------------------------------------------------SQCWRSRA   110
Cnas_D_XP_031624166        ----------------------------------------------------AQWSARRS   126
Dmel_hid                   AAFGWHGHP----HSPFTST--------------ST-------PLSAPVAPKMRLQRSQS   275
Hill_D_XP_037904604        ------------------------------------------------------RNGPPE   134
Bdor_D_AHX71830            I--FGATEATTHQRGCVATASTST-----------S-------TTSCGMGGHSRLHRSLS   244
Asus_D_AEF30451            V--FGSTEATTHQRTCVASTST-------------S-------TQSCMAKGHARLHRSFS   239
Ccap_D_JAB92755            NLFNGSTEATTHQRTCVASTSTAT----ASTSTAAS-------TSSCGAKGHARLHRSLS   258
Msca_D_APT68153            ------------------------------------------------------SLRSPR   99
Lser_D_AKA64736            S------------------------------------------TSGAGGMAHRSLHRSLS   215
Gfus_D_XP_037879387        --------------------------------------------GTSTGAVHRHLHRSLS   223
```

```
Cpip_D_XP_039434833      PLDVGGGG----------------------------------------GPS-GF--IPPH   308
Aead_D_XP_029727134      AAAAAGSGSSTGV-----SSSFNNGSSS------SSSSNSAGGSSAMVPMS-GC--IPPH   347
Bcop_D_XP_037040268      NEMPRRRLNSTGEEEDDEDIDSDIGIDECPSATSSSSNSSNSTPASPLPVDFNFEE---H   165
Bcop_D_XP_037050104      NGLR-RRLHSTGDDTHDDAESSD----------EFSMANGCNVPRQSVPVDYDLEE---H   156
Cnas_D_XP_031624166      VPRRRYRLTSAS-----DEEDYE-------D--EQP--------------LSDYEE---H   155
Dmel_hid                 DAARRKRLTSTG----EDEREYQ-------SDHEAT--------------WDEFGD--RY   308
Hill_D_XP_037904604      AVSRRSRLTSTG----DDDNEGH-------SEHETS--------------WDELDE--RY   167
Bdor_D_AHX71830          DAQKRSRRTST---TNDDEREYH-------SEHETS--------------WDEFDD--RY   278
Asus_D_AEF30451          DAQKRSRRTST---TNDDERECH-------SEHETS--------------WDEFDD--RY   273
Ccap_D_JAB92755          DAQKRSRRSSTN--ANDDERECH-------SEHETS--------------WDEFDD--RY   293
Msca_D_APT68153          TSTRRSRLTSTGDYYDDDDHECH-------SEHETS--------------WDELEEHHRL   138
Lser_D_AKA64736          DSARRSRLTSTG----EDEREYQ-------SEHETS--------------WDEFED--RY   248
Gfus_D_XP_037879387      DTARRSRLTSTG----EDEREYQ-------SEHETS--------------WDEYDD--RY   256


Cpip_D_XP_039434833      LDFMAGREQLQEFAGKIPQRKKKSK-----------------------------------   333
Aead_D_XP_029727134      LDYMAGREQLQEFAGKIPQRKKKSSSSK--------------------------------   375
Bcop_D_XP_037040268      YTYLAARERLQEFPGQVPQKKKKVVSEEKAEA----------------------------   197
Bcop_D_XP_037050104      YTYLAAREHLQPFSGQVPPKKKKRVVEHTEAD----------------------------   188
Cnas_D_XP_031624166      MQYMAERERLQVFPGRVPPPKKRDTTNTQ-------------------------------   184
Dmel_hid                 DNFTAGRERLQEFNGRIPPRKKKSSNSHSSSSNNPVCHTDSQPGGTSQAESGAIHGHISQ   368
Hill_D_XP_037904604      EQYMAARERLQEFPGRIPNRKKRKEGKK--------------------------------   195
Bdor_D_AHX71830          DNFTAGRERLQEFNGRIPPRKKKKDVPK--------------------------------   306
Asus_D_AEF30451          DNFTAGRERLQEFNGRIPPRKKKKDVPK--------------------------------   301
Ccap_D_JAB92755          ENFTAGRERLQEFNGRIPPRKKKKDVPK--------------------------------   321
Msca_D_APT68153          ENFTAGRERLQEFKGRIPPRKKKKEAPK--------------------------------   166
Lser_D_AKA64736          DNFTAGRERLQEFNGRIPPRKKKKDVPK--------------------------------   276
Gfus_D_XP_037879387      DNFTAGRERLQEFNGRIPPRKKKKDLPK--------------------------------   284
                           : * **:** * *::*  **:
```

```
Cpip_D_XP_039434833        ------AGGKSKKAGFLEQTLVWPTLAVTATLIALGCGLLAAR     370
Aead_D_XP_029727134        ------SSSKSKKSKFLEQTLVWPTLAVTATLIALGCGLLAAR     412
Bcop_D_XP_037040268        -----VAVKTQKKPKSKEQRFVWTTV-VTAAVLAVGYISSR--     232
Bcop_D_XP_037050104        ---------SPVAAKSKEQNFVWPTV-VTAVVLAVGCGFLVT-     220
Cnas_D_XP_031624166        -------------PRKSTDPKMWPAV-AAVVVLAVGCGFLVSR     213
Dmel_hid                   QRQVERERQKAKAEKKKPQSFTWPTV-VTVFVLAMGCGFFAAR     410
Hill_D_XP_037904604        ------TK---------EQSFTWPTV-VTVFVFAVGCGFLVAR     222
Bdor_D_AHX71830            ------SKYEKKVRERPFKGFIWPTV-VTVIVVAIGCGFLVTR     342
Asus_D_AEF30451            ------SKSEK----KQFKAFIWPTV-VTVIVVAMGCGFLVAR     333
Ccap_D_JAB92755            ------SRAER----KQFKAFIWPTV-VTVIVVAMGCGFLVAR     353
Msca_D_APT68153            ------TKPKAK-----DPCFTWPTV-VTVFVFAMGCGYLVAR     197
Lser_D_AKA64736            ------TKPEKK-----EQSFTWPAV-VTVFVFAMGCGFLVAR     307
Gfus_D_XP_037879387        ------PKSEKK-----EQSFTWPAV-VTVFVVAMGCGFLVAR     315
                                                 * :: .:. :.*:*
```
