# Supplementary material for: Deep Conservation of Hid-Like RHG Gene Family Homologs in Winged Insects Revealed by “Taxon Hopping” BLAST
Source: Insects. 2021 Oct 21;12(11):957. doi: 10.3390/insects12110957 (PMC8620624; doi:10.3390/insects12110957)
Supplement: Supplementary file 1 [file insects-12-00957-s001.zip › Supplementary data file S4_ MSA of lepidopteran RHG protein sequences.pdf]

```
Pxyl_L_XP_037962503         MAIAFNLPSEREASEENFSRLNRLLTELYEVLCHILVARQPEP---QERRDRPFSDSDIE  57
Hkah_L_XP_026316402         MAIAFNLPSEREATEENFSRLNRLLAELYEVLCHILVSRQPGQEAQENRRERPFSESDID  60
Herm_L_XP_021187231         MAIAFNLPSEREASEENFSRLNRLLAELYEVLCHILVSRQPGQEVQDNRRERPYSESDIP  60
Slit_L_XP_022815312         MAIAFNLPSEREATEENFSRLNRLLAELYEVLCHILVSRQPGQEVQENRRERPYSESDIP  60
Sfru_L_XP_035452846         MAIAFNLPSEREATEENFSRLNRLLAELYEVLCHILVSRQPGQEVQENRRERPYSESDIP  60
Atra_L_XP_013186325         MAIAFNLPSEREATEENFSRLNRLLAELYEVLCHILVSRQPGQEAQETRRDRPFSESDLD  60
Prap_LP_XP_022117210        MAIAFNLPSEREAAEENFSRLNRLLAELYEVLCHILVSRHPGQEVQDNRRDRPYSESDIV  60
Zces_LP_XP_038207226        MAIAFNLPSEREATEENFSRLNRLLAELYEVLCHILVSRQPGQEVQDNRRDRPFSESDIE  60
Ppol_LP_XP_013147603        MAIAFNLPSEREATEENFSRLNRLLAELYEVLCHILVARQPGQEVQENRRDRPYSESDIE  60
Dple_LP_XP_032522380        MAIAFNLPSERETAEENFSRLNRLLAELYEVLCHILVSRQPGQEVQENRRDRPYSESDVE  60
Vtem_LP_XP_026497378        MAIAFNLPSEREAAEENFSRLNRLLAELYEVLCHILVSRQPGQEAQENRRERPYSESDIE  60
Bmor_L_ICPK01046969         MAIAFNLPSEREAADENFSRLNRLLAELYEVLCHILVSRQPGE-VQENRRDRPHSDSELT  59
Msex_L_XP_030040660         MAIAFNLPSEREANDENFSRLNRLLAELYEVLCHILVSRQPGPEAQESRRERPHSDSEIL  60
                            ************: :**********:***********:*:*     : **:**.*:*::

Pxyl_L_XP_037962503         RPTFSGLPEVPPARRPGSVVFPPPALLNIDSEDLEDDVFVSPRFDNGNDLFSTRTMEIFG  117
Hkah_L_XP_026316402         RPGPAPQPQ--QCRRS---ASSSPPALTID-SNLEDDVFVSPRTANGNDLFSSRNLEIFG  114
Herm_L_XP_021187231         QP--APQTQ--QCRRS---ASSPPLTLTLD-SDLEDDV-FAPRSDRTNDLFATRNLDIFG  111
Slit_L_XP_022815312         QP--STQIQ--QCRRS---ASSPPLTLTLD-SDLEDDV-FAPRSDRTNDLFSTRNLDIFG  111
Sfru_L_XP_035452846         QP--TPQNQ--QCRRS---ASSPPLTLTLD-SDLEDDV-FAPRSDRTNDLFTTRNLDIFG  111
Atra_L_XP_013186325         RHNSPPP----QNRRS---ASSPPLTLTFD-SDLEDDVFVCPRTDNGNDPFSTTNLEIFG  112
Prap_LP_XP_022117210        RPSAAPP----PSRLS---ASSPPLTLPLD-MDLDDDVFVSPRTDNGNDRFSTTNLEIFR  112
Zces_LP_XP_038207226        RPSQAPP----FCRLS---ASSPPLTLPLD-IDLDDDVFVSPRTDNGNDPFSTTNIEIFR  112
Ppol_LP_XP_013147603        RP--APPPQ--PCRRS---ASSPPLTLTLD-SDLEDDVFVSPRTNSGSDPFSTTNLEIFR  112
Dple_LP_XP_032522380        RQTQASPQQ--HCRRS---ASSPPLTLTFD-S-DLEDDVFSPRTDAGNDRFSTTNLEIFR  113
Vtem_LP_XP_026497378        RQQISTP-Q--QCRRS---ASSPPLALTFD-SDDLEDDVFAPRTDNGNDRFSTTNLEIFN  113
Bmor_L_ICPK01046969         I---SRNPQ--PARLS---ASSPPLSLTFD-SDIEDDVF-APRTDR-NDIFSTRNVEIYG  108
Msex_L_XP_030040660         TR--PSTSQ--PCRLS---ASSPPLSLTFD-TDLEDDVFTAPRSDR-NDIFSTRNVDIFG  111
                                         *     .   *  * :*     :*   .**    .* *:: .::*:
```

```
Pxyl_L_XP_037962503         SRFGNPRTPAGVLLSPRRERRFTFAVTSEKHWRDKGGTREEEDAGIPISDTDEPDAPSPE  177
Hkah_L_XP_026316402         --YGSPRTPAGVLLNGRSERSFTFSVSSQKQCRDKGGTDHADGA----HAAYTDDDDAN-  167
Herm_L_XP_021187231         --YGSPRTPAGVLLSPRRERTFTFSVTSEKHWRDKGGTDVADGV----SRGDGISDTDSD  165
Slit_L_XP_022815312         --YGSPRTPAGVLLSPRRERTFSFSMTSEKHWRDKGGTDVADGV----TPGELSDAECDR  165
Sfru_L_XP_035452846         --YGSPRTPAGVLLSPRRERTFSFSMTSEKHWRDKGGTDVADGV----TPDEMSDAECDR  165
Atra_L_XP_013186325         --YGSPRTPAGVLLSPRRDRKFTFSVSSEKHWRDKGGTDVADGV----TDADVTDAVTS-  165
Prap_LP_XP_022117210        --YGSPRTPAGVLLSPRRESSFNFSVTSEKHWRDKGGTDVADGV----GDTDETDAASP-  165
Zces_LP_XP_038207226        --YGSPRTPAGVLLSPRRDSSFSFL-TSEKHWRDKGGTDVADGI----MDTDVTDAALP-  164
Ppol_LP_XP_013147603        --YGSPRTPAGLLLSPRRERAFTFSVTSEKHWRDKGGTDVADGV----ASADVTDSSSP-  165
Dple_LP_XP_032522380        --YG--RTPAGVLLSPRRERSFTFSVTSEKHWRDKGGTDVADGV----VDEDVTDAADP-  164
Vtem_LP_XP_026497378        --YG--RSPAGVLLSPRRERSFNFSVTSEKHWRDKGGTDVVDNV----IDADVTDAATP-  164
Bmor_L_ICPK01046969         --CNSPRTPGGVLFSPRRERKFTFSS-SEKHWRDKGGTDVTDGV----SDADETDAGTPD  161
Msex_L_XP_030040660         --YGSNRTPAGVLLSPRRERSFTFSSSSEKHWRDKGGTDVADGV----TDTDETDAISPE  165
                               .  *:*.*:*:. * :  *.*   *:*: ******   :.           .

Pxyl_L_XP_037962503         -----PSSSPKDSTSSESSIFSSIMPDKLSGSICTAVLFMLGWKLLANNR     222
Hkah_L_XP_026316402         --AASNENQQDERGSSVSSVISSITPYKVSGSICTAILLMLGWTILSHKR     215
Herm_L_XP_021187231         RDLDETDAPPT-EKAAETSIFSSIIPDKLSGSICTAVLFVLGWKLFSNKR     214
Slit_L_XP_022815312         DNMDETDAPKAEEKEPEGSIFSSIIPDKLSGSICTAVLFMLGWKLFSNKR     215
Sfru_L_XP_035452846         DILDETDAPKPEEKEPEGSIFSSIIPDKLSGSICTAVLFMLGWKLFSNK-     214
Atra_L_XP_013186325         ------PTSS----EKSEGSISSIIKDKLPGSICTVVLFMLGWKLLSNKR     205
Prap_LP_XP_022117210        ------DCPND-KRSSESSILSSIMPDSISGSICTAVLFMLGWKLLSNKR     208
Zces_LP_XP_038207226        ------QCTDE-KRSSESWIFSSIIPDKLSGSICTAVLFMLGWKLLSNKR     207
Ppol_LP_XP_013147603        ------EEPYQPEKSSSESIFSSIIPDKLSGSICTAVLFMLGWKLLANKR     209
Dple_LP_XP_032522380        ------EPPFAGKKESENSILSSILPDKLSGSICTAVLFVLGWKLLSNKR     208
Vtem_LP_XP_026497378        ------RLTEKETSSS-ESSIFSILPDKLSGSICTAVLFMLGWKLFSNKR     207
Bmor_L_ICPK01046969         A-------PT-STEPGVTSILTSIIPDKLSGSICTAVLFMLGWKLFSKR-     202
Msex_L_XP_030040660         D-------PA-QKD-SSTSIFSSIIPDKLSGSICTAVLFMLGWKLFSQR-     205
                                                : **   .: *****.:*::***.::::.
```
