# Supplementary material for: Deep Conservation of Hid-Like RHG Gene Family Homologs in Winged Insects Revealed by “Taxon Hopping” BLAST
Source: Insects. 2021 Oct 21;12(11):957. doi: 10.3390/insects12110957 (PMC8620624; doi:10.3390/insects12110957)
Supplement: Supplementary file 1 [file insects-12-00957-s001.zip › Supplementary data file S5_ MSA of coleopteran RHG protein sequences.pdf]

```
Tcas_C_XP_008194456.1        MAVPFVLPEDEINNLIVAV--IFILS----QTRLQRNEENRELQE-----------CMEE 43
Atum_C_XP_019872614.1        MAVPFNMPEDHNEVDVIVNQLQSLLT-QLQASVRESQRSTRTRNP---------PGSTRH 50
Rfer_C_KAF7270649            MAVPFIIPDDQNDSQVILVQLQTLIS-RLQMIIHNQQQQQQLSVN------ASSNSYNNS 53
Sory_C_XP_030755961          MAVPFTMPDDQNEVEVLLIQLQALLT-KLQIAARKHRLRSQQAQSNGSAAVATLTTTAPL 59
Dpon_C_XP_019765467.1        MAVPFNIPEDQSEVEVLLIQLQTILN-KLQATVRQNHQLAQ-QP--------TASIPSGS 50
Dvir_C_XP_028151061.1        MAIPFNLPGEQTEVEVIITQLQYLLM-KLQDAIRRSGATNSSYP--------VFSISTRT 51
Ldec_C_XP_023021532.1        MAVPFVMPEDENEVEVIIIQLQSLLT-KLQAALRRSRGPPPPRT--------SLPT---R 48
Agla_C_XP_018573717.1        MAVPFNMPEDQNEVEVIINQLQSLLT-KLQAAVRRSRGPTIRTN--------QTAV---T 48
Obor_C_KRT86088.1            MAIPFTIPNQEVSQLN-IQELLRELCEILQQLLIICRQQSINQQQ-----------QQAT 48
Otau_C_XP_022915163.1        MAIPFAVPGQPPV----SPERLRELYDFLQQLLIVRQQQNHHRQE-----------EVVT 45
Nves_C_XP_017785459.1        MAIPFDVLNDEEGDPA-LNELLSQLCDAIICLRRVHSH---IQFA--------------- 41
Aplani_C_XP_018330969.1      MAIPFDVSSDEK-----LDQLFTRLYEILQQIYHLTQQTASGQQP-----------TI-T 43
Ppyr_C_XP_031336702.1        MAIPFNVENDSDCDEAVLQLLLSDLCRTLQQIALFQRSRRTQNLN-----------RAPS 49
Ilum_C_KAF2880729.1          MAIPFLVEDDSEYEEALLQQLYSDLFHTIQQIELIQRSRRAQHQI-----------SPRV 49
Ater_C_KAF5276935.1          MAVPFTVEDESDNYEIVLQQLFSDLRQTLQQIVDMQRLRRTQNLL-----------ATTR 49
                             **:** :  :              :

Tcas_C_XP_008194456.1        LVRLLAEELQRRSRS---------------SEYDNQLKQTL-----LLLRQS-------- 75
Atum_C_XP_019872614.1        RVRFASGTRD-RDPERQEET------DGDGRPSHEHLADLM---------VCRHDVPSAS 94
Rfer_C_KAF7270649            RVRSSSTTRQ-RTRDSSE--------TSSTTPNQNNLSEVISN--LSLFALSAGDQQSSS 102
Sory_C_XP_030755961          RVRGSSINRQ-RSESSET--------AASPAQTTNAIPDLISN--LSWLASSHNETQAAS 108
Dpon_C_XP_019765467.1        RVRYSPDDRV-RSREVLQ------SVERITPVPQNVLPDLLSN--LSWLADSSNEASSSS 101
Dvir_C_XP_028151061.1        RVRTNSSNRA-RGREQSSVSSPETPSTSASPSANANLPELLNN--LAWLAVLTQEAVDNN 108
Ldec_C_XP_023021532.1        NRTSAGSSRS-PTQDQGT--------STSASAPSTQIPDLLNN--LALLAAASQEIVQSN 97
Agla_C_XP_018573717.1        RVRSPASSRL-RSRD-ES--------SATTSISSTNFPDLLNN--IAWLAAASQDAASTS 96
Obor_C_KRT86088.1            RNRASTSSVV--NQH-------------------DPIPEIMTN--LSLLSAA-------- 77
Otau_C_XP_022915163.1        TRREDTTSVR--ASG-------------------RAADG-------RMLSAL-------- 69
Nves_C_XP_017785459.1        ----SRGSSL-NPQRI--------------REAFGPHPNVLERLSRNLLPLPDVVGERTD 82
Aplani_C_XP_018330969.1      RVRCSAGASR----HIQD--------HQIHSSYPAVLPEIVND--LALLPRPSAQYLEGE 89
Ppyr_C_XP_031336702.1        SP--ATTTS----RHSGA--------S---SETERTLLNSTVD--LALSLSQ-------V 83
Ilum_C_KAF2880729.1          RTTPSAGTSR-RNREVPG--------DPLDSENLAPEPVVNLA--LALSPAGSQNDL--D 96
Ater_C_KAF5276935.1          AT-----SIR-RPREHGT--------QTEFSEA---LFNSTVN--LALSPAGAQNGL--E 88
```

```
Tcas_C_XP_008194456.1        ------LSHFTPTLSR-IQRRSNFESCCDDCKISLH-----------EDCATKDCSQVGL 117
Atum_C_XP_019872614.1        ---------TQLTASPIRRRTTRFQTDCDETKINIT-------TIVDT-GSQQDWAQIGI 137
Rfer_C_KAF7270649            ------TYTAIPVRSPRTRG-CSESSNCDVTKTATK-------T-----TFDQDWNKMNC 143
Sory_C_XP_030755961          ------ALTAMPVRSPRRRRTNSESSNCDKTTKTTT-------T-----DLSQDWNKVNC 150
Dpon_C_XP_019765467.1        SPL-----T-AMSARSRRRRTASESSNPDETKISVT-------AIVTTDFCAQDWNEKNC 148
Dvir_C_XP_028151061.1        SQ----QTTAMAVPRPTRNRTSSESSCCQETKSDET-------TASNS-GSVQAWNQETD 156
Ldec_C_XP_023021532.1        QPEGAPQVAASATFRSTRRRTNSESSCCEESKVSIT-------TA-NR-NGVQDWNQVNE 148
Agla_C_XP_018573717.1        S-----SLTANSVTRSPRRRTSSESSNCKETKISIT-------AI--V-NHAQDWNQADG 141
Obor_C_KRT86088.1            --FNSIQTTPARALRRPRRYRLSSESSCDETKNFDHQTTSTTTSPTTSAQISPEWMEVDM 135
Otau_C_XP_022915163.1        --SDA-----LHTSRRPRRRWPKSPTHH---------DDSSNSNSAAKEAQNQRWKRVGT 113
Nves_C_XP_017785459.1        -VSL--QTIEASAIPGRRQRTTSESSCCKVPKIANM--------QTPIDQPQ-DYSEDDD 130
Aplani_C_XP_018330969.1      -AVLQPQ--AMPTPRRRKRRRTSSKSCCDANKSVEA--------ATVEDDGHQVWTQVGM 138
Ppyr_C_XP_031336702.1        -EALQQQNASVSTRRRHPRRSRSEPSCCKEPKPVVI--------SVAVEVTNKDWIQKEH 134
Ilum_C_KAF2880729.1          -AILRPQNLSTPTRRRRRRRTRSEPSCCEENKPVKV--------SVSVEVTNKEWNQQEK 147
Ater_C_KAF5276935.1          -MLLQLESLFLPVRRLHRRRSQSEPPFCKEVKPVKV--------SVSVEVGNKDWIQQEQ 139
                                               .                                     .

Tcas_C_XP_008194456.1        ILRKIDDDETSAIFKEGVEEE------------------KEGFVEESLLSRLVPRVR--- 156
Atum_C_XP_019872614.1        TLRNIADDINPVTFREVV---------------------LFPTTKRGLLSKL-PDALT-C 174
Rfer_C_KAF7270649            NNGDDDDDDDDDE--------------------------NKEASSVGLLSKLTPASVSSC 177
Sory_C_XP_030755961          DDDGDD----------------------------------EGGSSDGLLSKIMPATVSSC 176
Dpon_C_XP_019765467.1        VD--------SGT--------------------------SNVIPADGLFSKLIPGPVSSC 174
Dvir_C_XP_028151061.1        NEQSSE----DGS--------------------------DKSYSRQGILSRLIPGPVS-A 185
Ldec_C_XP_023021532.1        PE--------ISS--------------------------AKTLSSESIFSKFVPGPVS-A 173
Agla_C_XP_018573717.1        SP--------DSE--------------------------PEQASGDGILSKFVPGPVS-A 166
Obor_C_KRT86088.1            DLRKISEDFKAAGFHKDDDAEEEDTTALQSSSKSSSSSPSTSTKRDSIFSKFVPAPF-TG 194
Otau_C_XP_022915163.1        ELRKIADEFESAAFEEVDDVDEVDSSLP------CCSRLSTSKERKGTFSKILSMPVMVS 167
Nves_C_XP_017785459.1        -----------------------P--------SSQRPKTRTACDGDGLLSTLVPSPI-KT 158
Aplani_C_XP_018330969.1      PLLKMAKDFDPSEQQFTCKEK-ND--------DEVTGAKEDSCQSESIFSKLVPDLS-SK 188
Ppyr_C_XP_031336702.1        --------------------------------HISPTTKKPWYRSDGLLSKLIPAQL-SG 161
Ilum_C_KAF2880729.1          --------------------------------SSQSSPDKQSCGSESLLSKLVPAPF-SG 174
Ater_C_KAF5276935.1          --------------------------------STETSTKKKWCGSENLLSKLVPAPF-SG 166
                                                                           . :* :
```

```
Tcas_C_XP_008194456.1           TFWKAVPLICVVGLHLFQISYSM 179
Atum_C_XP_019872614.1           TIWTA--MLGCVGWHLFRSR--- 192
Rfer_C_KAF7270649               SLMTA--VICFVGWHIFRGR--- 195
Sory_C_XP_030755961             SFMTA--MICFVGWHIFCGR--- 194
Dpon_C_XP_019765467.1           SLMTA--MICFVGWHIFRAR--- 192
Dvir_C_XP_028151061.1           PLFTA--MICFVGWHIFRAR--- 203
Ldec_C_XP_023021532.1           PLWTA--MICFVGWHLFGTR--- 191
Agla_C_XP_018573717.1           PLWTA--MICFVGWHLFRAR--- 184
Obor_C_KRT86088.1               SICTA--VICVVGWHLLRNR--- 212
Otau_C_XP_022915163.1           SICTA--VLC-VGWHLLRNR--- 184
Nves_C_XP_017785459.1           SIWTA--VICAVGWYMFRAR--- 176
Aplani_C_XP_018330969.1         SIWTA--VICVVGWHLLRTR--- 206
Ppyr_C_XP_031336702.1           PLWTA--VICMVGWHLLRTR--- 179
Ilum_C_KAF2880729.1             PLWTA--VICVVGWHLLRTR--- 192
Ater_C_KAF5276935.1             PLWTA--VICVVGWHLLRTR--- 184
                                 : .*  ::  ** :::
```
