# Supplementary material for: Deep Conservation of Hid-Like RHG Gene Family Homologs in Winged Insects Revealed by “Taxon Hopping” BLAST
Source: Insects. 2021 Oct 21;12(11):957. doi: 10.3390/insects12110957 (PMC8620624; doi:10.3390/insects12110957)
Supplement: Supplementary file 1 [file insects-12-00957-s001.zip › Supplementary data file S6_ MSA of hymenopteran RHG protein sequences.pdf]

```
Nlec_HY_XP_015511746        MAIPFALPSEDEAE-AQE-RRLQGLLLDVYGILQQVIRARSYQNPG-QNHRVNRRSGEVP      57
Nvit_HY_XP_003424554        MAIPFALPSDEDAE-DQSQQQLEQLMLDLYAALQQVLRARNSQHPS-RNHRLPRRSADMS      58
Btre_HY_XP_033214430        MAIPFALPSEDEAE-AQG-QQLEQLMLDLYAALQQVLRNRSLQHPS-RNHRLPRRSGDIS      57
Mdem_HY_XP_008555096        MAIPFNLPSEDEAE-AQS-QQLEQLMLDLYAALQQVLRSRSAQHPS-RNHRLPRRVGDTG      57
Ccin_HY_XP_015609297        MARPFTLPSEDEAE-EQG-QQLEQLMLDLYAALQQVLRSRRAEHPS-RNHRLPRRAGDVP      57
Oabi_HY_XP_012277082        MAIPFALPSEDEAE-AQG-QQLEQLMLDLYAALQQVLRIRNAQHPS-RNHRLPRRSGDMP      57
Ccal_HY_XP_017884183        MAIPFALPTEDDAE-AQG-QQLEQLMLDLYAALQQVLRTRTSQHPSSRNHRIPRRA----      54
Bimp_HY_XP_024222863        MAIPFILPSDDEAE-VQG-QDLEQLMLALYAALQQVLRNRNDQHPS-RNHRLPRRM----      53
Bter_HY_XP_012175674        MAIPFILPSDDEAE-VQG-QELEQLMLALYAALQQVLRNRNDQHPS-RNHRLPRRM----      53
Dnov_HY_XP_015429159        MAIPFSLPSDDDAE-AQG-QQLEQLMLDLYAALQQVLRSRTAQHPS-RNHRLPRRAGD--      55
Nmel_HY_XP_031834337        MAIPFSLPSDDDAE-AQG-QQLEQLMLDLYAALQQVLRSRTAQHPS-RNHRLPRRAGDGF      57
Mgen_HY_XP_033321541        MAIPFSLPSDDDAE-AQG-QQLEQLMLDLYAALQQVLRSRTAQHPS-RNHRLPRRAGDGF      57
Ador_HY_XP_006609937        MAIPFALPSDDEAE-TQT-QQLEQLMLDLYAALQQVLRSRTAQHPSSRNHRLPRRS----      54
Aflo_HY_XP_012338864        MAIPFALPSDDEAE-TQT-QQLEQLMLDLYAALQQVLRSRTAQHPSSRNHRLPRRS----      54
Amel_HY_XP_006565903        MAIPFALPSDDEAE-TQT-QQLEQLMLDLYAALQQVLRSRTAQHPSSRNHRLPRRS----      54
Olig_HY_XP_034171762        MAIPFALPSDDEAE-AQG-QQLEQLMLDLYAALQQVLRSRTVQHPS-RNHRIPRRAADGF      57
Obic_HY_XP_029055907        MAIPFALPSDDEAE-AQG-QQLEQLMLDLYAALQQVLRSRTVQHPS-RNHRIPRRAADGF      57
Emex_HY_XP_017765203        MAIPFVLPSNDEAE-AQG-QQLEQLMLDLYAALQQVLRSRNTQHPS-RNHRLPRRV----      53
Pgra_HY_XP_020280259        MAIPFTSERS----------PEEQVLLDIYLCIENALRSRSARHP-SRNHRAPRQHVGI-      48
Obir_HY_XP_011342863        MAIPFVQNDDNNNERSQLEEQQLKLMFDLYVSLHNVLRSRHAQHTASRNHRVPRQPAGTN      60
                            ** **    .              ::: :*  :.:.:* *  .:   :***  *:

Nlec_HY_XP_015511746        GPREPPVVLV----DSDLSRLPLPDLVSSAI---EQLPL-PMPQVTASAPSSPTRDVAFQ      109
Nvit_HY_XP_003424554        GTRDHPMLLV----DSDLSRLPLPDLVLSAV---EQLPA-PPPQVTVSAPSSPTRDAAFQ      110
Btre_HY_XP_033214430        SPREHPIVLV----DSDLSRLPLPDLVLSAV---EQLPP-PTPQVTVSAPSSPTRDAAFQ      109
Mdem_HY_XP_008555096        VSRDHPIVLI----DSDLSRLPLPDLVLSAV---QQLPS-PEPQVTVSAPSSPTRDAAFQ      109
Ccin_HY_XP_015609297        GSREHPVVLV----DSDLSRLPLPDLVLSAV---EQLPP-PTPLVTVSAPSSPTRDAAFQ      109
Oabi_HY_XP_012277082        GFREHPVVLI----DSDLSRLPLPDLVLSAV---EQLPT-PTPLVTASAPSSPTRDAAFQ      109
Ccal_HY_XP_017884183        GDERHPMVLL----DSDLSRVPLPDLVLSAV---EQLPP-PTPQVTVSAPSSPTRDAAFQ      106
Bimp_HY_XP_024222863        GDDRHPMVLV----DSDLARMPLPDLVLSAV---EQLPP-AAPQVTVSAPSSPTRDATFQ      105
Bter_HY_XP_012175674        GDERHPMVLV----DSDLARMPLPDLVLSAV---EQLPP-AAPQVTVSAPSSPTRDATFQ      105
Dnov_HY_XP_015429159        -SREHPVVLI----DSDLSRLPLPDLVLSAV---EQLPA-ATPQVTVSAPSSPTRDVAFQ      106
Nmel_HY_XP_031834337        GSREHPVVLV----DSDLSRLPLPDLVLSAVEQVEQLPA-ATPQVTVSAPSSPTRDAAFQ      112
Mgen_HY_XP_033321541        GSREHPVVLV----DSDLSRLPLPDLVLSAVEQVEQLPA-ATPQVTVSAPSSPTRDAAFQ      112
Ador_HY_XP_006609937        GDDRQLVVLV----DSDLSRLPLPDLVLSAV---EQLPP-PTPQVTVSAPSSPTRDAAFQ      106
Aflo_HY_XP_012338864        GDDRQLVVLV----DSDLSRLPLPDLVLSAV---EQLPP-PTPQVTVSAPSSPTRDAAFQ      106
Amel_HY_XP_006565903        GDDRQLVVLV----DSDLSRLPLPDLVLSAV---EQLPP-PTPQVTVSAPSSPTRDAAFQ      106
Olig_HY_XP_034171762        GSREHPVVLV----DSDLSRLPLPDLVLSAV---EQLPA-ATPQVTVSAPSSPTRDAAFQ      109
Obic_HY_XP_029055907        GSREHPVVLV----DSDLSRLPLPDLVLSAV---EQLPA-ATPQVTVSAPSSPTRDAAFQ      109
Emex_HY_XP_017765203        GDERHPVVLV----DSDLSRLPLPDLVLSAV---EQLPA-ATPQVTVSAPSSPTRDAAFQ      105
Pgra_HY_XP_020280259        --PQPVVIWLPSGNVEELSSVPLPDLVQSTM---EQLPP-PSPQVTASVPSSPMRDATFQ      102
Obir_HY_XP_011342863        NPQQPVVIVLHPGNAEDMSTLPLPDLVQSAM---DQLPPPPSPHVTVSVPSSPMRDATFQ      117
                                  :: :     .::: :****** *::   :***    * **.*.**** **.:**
```

```
Nlec_HY_XP_015511746        FPELNP-----SAPVPSPRPRPRRRRLVSENSALKKEQPST-----------------SY      147
Nvit_HY_XP_003424554        FPECSGASAAMMMPVPSPRPKPRRRRLTSEGSLPEKAQLPQRLDSCTGGRGTCCAHHL--      168
Btre_HY_XP_033214430        FPECS-------APVPSPRPKPRRRRLTSESSIPEKGPSES----NK-CNGTCCPGHLNL      157
Mdem_HY_XP_008555096        FPECSA------APVPSPRPRPRRRRLTSESSVLEKGATSR----SDNINSVCC-RHL--      156
Ccin_HY_XP_015609297        FPECSA------APVPSPRARPRRRRLTSEGSVLEKER-----------VNASC-GQI--      149
Oabi_HY_XP_012277082        FPEAS-------VPVPIPRPRPRRRRLTSECSVQEKER-----------MNACC-GQP--      148
Ccal_HY_XP_017884183        FPECSGES--SLAPVPSPRPRPRRRRLASESSGAATAKKTP--PAAKQICNNCF-GDL--      159
Bimp_HY_XP_024222863        FPESSNDVN-DVAPVPNPRPRPRRRRLASEGGSSAAVKKT---SAVKSICKCCC-GHI--      158
Bter_HY_XP_012175674        FPESNNDAI-DVAPVPNPRPRPRRRRLASEGGSSAAVKKT---SAVKSICKCCC-GHI--      158
Dnov_HY_XP_015429159        FPECSTDE----APEPSPRPRPRRRRLASEG-G--AVKKG---PIGEPTCNSCC-GHF--      153
Nmel_HY_XP_031834337        FPECSSDE----APVPSPRPRPRRRRLASEG-G--AVKKG---PVGESACNNCC-GHF--      159
Mgen_HY_XP_033321541        FPECSNDE----APVPSPRPRPRRRRLVSEGSG--AVKKG---PVGELTCNSCC-GHF--      160
Ador_HY_XP_006609937        FPECSGDEN-GAAPVPSPRPRPRRRRLASEGGG-ATMKKG---PATDRICKKCC-GHL--      158
Aflo_HY_XP_012338864        FPECSGDEN-GAAPVPSPRPRPRRRRLASEGGG-ATTKKG---PATERICKKCC-GHL--      158
Amel_HY_XP_006565903        FPECSGDEN-GAAPVPSPRPRPRRRRLASEGGG-ATMKKG---PATERICKKCC-GHL--      158
Olig_HY_XP_034171762        FPECSGDEN-VLAPTPNPRPRPRRRRLASECGGAAAMKKG---P-AEPTCNNCC-GHF--      161
Obic_HY_XP_029055907        FPECSGDEN-VLAPTPNPRPRPRRRRLASECGGAAAMKKG---P-AEPTCNNCC-GHF--      161
Emex_HY_XP_017765203        FPECSGDES-GLAPVPSPRPRPRRRRLTSEGGSAAAMKKG---PVAEPICNSCC-GHY--      158
Pgra_HY_XP_020280259        FPECSVNGN--IVPLPSPRPLPRR-RLASEGSSLRTLRS-ETRQNEGNTSNTCCC-YP--      155
Obir_HY_XP_011342863        FPECTN-SS--SGPLPSPRPRPRR-RFASESSIMESSVMGPSGPIEVSICNGSCC-HL--      170
                            *** .        * * **  *** *:.**

Nlec_HY_XP_015511746        YQAPSDENEWANVGANLRNIADDFHASKNTDSGAEKSSLPVIGND---------------      192
Nvit_HY_XP_003424554        -AASLDEREWATVGANLRNIADDFHASKTKDAEVEKSRLPFIGSSFSSSTSGLGSSGLGS      227
Btre_HY_XP_033214430        SATTLNEREWVAVGTNLRNIADDFHASKTKDAEVEKSRLPLLGSTFSSSTS---------      208
Mdem_HY_XP_008555096        -GHSCNETEWASVGANLRNIADDFHASKTKDAEIEKSRLPFFGAGFPSVTTS--------      207
Ccin_HY_XP_015609297        -GTVVNEREWTSVGTNLRNIADDFHAAKTKDAEVEKSRLPFIGSGFSS------------      196
Oabi_HY_XP_012277082        -PTA-IEREWASIGSNLRNIADDFHASHTKGAEVEKSRLPFIGSTS--------------      192
Ccal_HY_XP_017884183        HTQAYNQNQWTNMGANLRNIADDFHASKTKAEEIEKSRLAAMNPGYASSGN---------      210
Bimp_HY_XP_024222863        -GQAFNGNYWATVGANLRNIADDFHASKTKAAEIEKSRLSAMNPSFANTNN---------      208
Bter_HY_XP_012175674        -GQAFKGSYWATVGANLRNIADDFHASKTKAAEIEKSRLSAMNPSFANTNN---------      208
Dnov_HY_XP_015429159        -GQAFNESQWASVGANLRNIADDFHASQTKAAEIEKSRLPALSPGFASAQN---------      203
Nmel_HY_XP_031834337        -GQAFNESQWASVGANLRNIADDFHASKTKAAEIEKSRLPALSPGFAGANN---------      209
Mgen_HY_XP_033321541        -GQAFNESQWASVGANLRNIADDFHASKTKAAEIEKSRLPTLSPGFVGSNP-------AA      212
Ador_HY_XP_006609937        -GQAFNESQWASVGANLRNIADDFHASQTKAAEIEKSRLPAMNPSFADSNN---------      208
Aflo_HY_XP_012338864        -GQAFNESQWASVGANLRNIADDFHASQTKAAEIEKSRLPAMNPSFADSNN---------      208
Amel_HY_XP_006565903        -GQAFNESQWASVGANLRNIADDFHASQTKAAEIEKSRLPAMDPSFADSNN---------      208
Olig_HY_XP_034171762        -GQAFNESQWANVGANLRNIADDFHASKTKAAEIEKSRLPAMNPGFANSGN---------      211
Obic_HY_XP_029055907        -GQAFNESQWANVGANLRNIADDFHASKTKAAEIEKSRLPAMNPGFANSGN---------      211
Emex_HY_XP_017765203        -GQAFNESQWASVGANLRNIADDFHASKTKAAEIEKSRLPAMNSGFANSSN---------      208
Pgra_HY_XP_020280259        -S-QNERQSWTNMGTNLRNIAGDFHACKTKDAESEKSRLSVNGRRLTNG-----------      202
Obir_HY_XP_011342863        -G-QNERQSWANVGTNLRSIAGDFHACRTKDAEGEKSRLPVIGPGLPNARN---------      219
                                     *. :*:***.**.****.:..    *** *   .
```

```
Nlec_HY_XP_015511746        --ESSPDSFLSLLIPAPLRETLWTTVALYLGWRLVSHLR  229
Nvit_HY_XP_003424554        SPSGSTDSILSLLMPAPLRETLWTTLALYLGWRLVSRLR  266
Btre_HY_XP_033214430        --NSSTDSILSLLIPAPLRETLWTTVALYLGWRLVSRLR  245
Mdem_HY_XP_008555096        --IPSSDGFLSMLIPAPLRETLWTTVALYLGWRLVSRLR  244
Ccin_HY_XP_015609297        --SPSTDSLLSLLIPAPLRETLWTTVALYLGWRLVSRLR  233
Oabi_HY_XP_012277082        ---SAADSFFSFLLPAPLRETLWTTVALYLGWRLVSRLR  228
Ccal_HY_XP_017884183        ---SSTDSYMSLLILAPLRETLWTTVALYLGWRLVSHLR  246
Bimp_HY_XP_024222863        -SSESTDSFLSLLIPAPLRETLWTTVALYLGWRLVSRLR  246
Bter_HY_XP_012175674        -TSGSTDSFLSLLIPAPLRETLWTTVALYLGWRLVSRLR  246
Dnov_HY_XP_015429159        -TNGSTDSLLSLLIPAPLRETLWTTVALYLGWRLVSRLR  241
Nmel_HY_XP_031834337        -TNGSTDSILSLLIPAPLRETLWTTVALYLGWRLVSRLR  247
Mgen_HY_XP_033321541        SNAGSTDSLLSLLIPAPLRETLWTTVALYLGWRLVSRLR  251
Ador_HY_XP_006609937        -TSGSTDSILSLLIPAPLRETLWTTVALYLGWRLVSRLR  246
Aflo_HY_XP_012338864        -TSGSTDSILSLLIPAPLRETLWTTVALYLGWRLVSRLR  246
Amel_HY_XP_006565903        -TSGSTDSILSLLIPAPLRETLWTTVALYLGWRLVSRLR  246
Olig_HY_XP_034171762        -ATGSTDSLLSLLIPAPLRETLWTTVALYLGWRLVSRLR  249
Obic_HY_XP_029055907        -AAGSTDSLLSLLIPAPLRETLWTTVALYLGWRLVSRLR  249
Emex_HY_XP_017765203        -ANGSTDSILSLLIPAPLRETLWTTVALYLGWRLVSRLR  246
Pgra_HY_XP_020280259        -NSNSTTDMLSLLIPTPLRETLWATVALYVGWKLVSRLR  240
Obir_HY_XP_011342863        RNSSFTDNLLSLLIPTPLRETLWATMALYLGWRLVSRLR  258
                                   . :*:*: :*******:*:***:**:***:**
```
