# Supplementary material for: Deep Conservation of Hid-Like RHG Gene Family Homologs in Winged Insects Revealed by “Taxon Hopping” BLAST
Source: Insects. 2021 Oct 21;12(11):957. doi: 10.3390/insects12110957 (PMC8620624; doi:10.3390/insects12110957)
Supplement: Supplementary file 1 [file insects-12-00957-s001.zip › Supplementary data file S7_ MSA of hemipteran RHG protein sequences, Aphidoidea excluded.pdf]

```
Clec_HE_XP_014239966.1        MAVPFNVPEEEAEERRVIRIP-----ICEFIRQFIQPE-----ISNLQPENP--------  42
Btac_HE_XP_018898908.1        MAIPFNLPSDEETEDHYKRLDQLLKDLYDVLRQILFRPRPIHDDD---------------  45
Lstr_HE_RZF36208.1            MAIPFNLPSEEDTEEYYRRLDQLLADLYDVLRHILFRPRTQQPAHNHQQQQA-PVVDAHG  59
Nlug_HE_XP_022189933.1        MAIPFNLPSEEDTEEYYRRLDQLLADLYDVLRHILFRPRTQQPAHNHQQ-QP-PVIDADG  58
Btac_HE_XP_018895589.1        MAIPFHLPSDEETEENYRRLDQLLADLYDVLRQILLRPRHMQAPSTRIVENTGFTTESE-  59
Aluc_HE_KAF6204998.1          MAIPFNLPSDEETEDAYRRIDQLLLDLYDLLRQIILRQRPVQNERGVSEARISPTWD-E-  58
Ofas_HE_GCXY01041673.1        MAIPFNWPSDEETEDHYRRLDQLLLDLYDVLRQILLRRRLHPVPQPE--AQPVPLHEHL-  57
Hhal_HE_XP_014287364.1        MAIPFNLPSDEETADNYRRLDQLLLDLYEVLRQILLRRRIERVAGPI--ERY--------  50
                              **:**: *.:*       *:      : :.:*:::

Clec_HE_XP_014239966.1        -----------------RPE--GPVILVLPEAVLRA------------------------  59
Btac_HE_XP_018898908.1        ------------------LTGFEARLLALPDLVQSAMMSNYPPAATATAHSPWSAIGARD  87
Lstr_HE_RZF36208.1            DNESEDHEPPDHGGEDPSPDALEQRLHPLPDLVANVGSSSAAP-SR--------------  104
Nlug_HE_XP_022189933.1        DNESEDQEPPDHGGEDPSPDALEQRLHPLPDLVANVGSSSAAPSSR--------------  104
Btac_HE_XP_018895589.1        ----------QDFEPPSDSDLVQERLMALPDLVRSAMSPALNNLSNRLQESNLEC-----  104
Aluc_HE_KAF6204998.1          ----------QAASMATQTVETTRLESRLPDLIADTLPSLVANTRQSL------------  96
Ofas_HE_GCXY01041673.1        ----------QPQGLQRQQLFRQARRPDLPDLIPSAA-------NPIA------------  88
Hhal_HE_XP_014287364.1        ----------------PQQPQPQPQQPGLPDLVP--------------------------  68
                                                          **: :

Clec_HE_XP_014239966.1        ---------RKAPAAPRRRRRRQ-------------------------------------  73
Btac_HE_XP_018898908.1        DAADETTNGSRHCRHRHCHTPKRVDLTVAT-VARVDAGGGGHLHLRPPAPPPRPPPGSQS  146
Lstr_HE_RZF36208.1            ------PLGRGHPRRHQDDRRRRRDSTT---TPDDSRLSP---EDQR-PPPPRKPRESKK  151
Nlug_HE_XP_022189933.1        ------PLGRGHPRRRRRDSTSDSVTINNSRMTSNSRTTDDEEVVDP-PRPLRKLRESKK  157
Btac_HE_XP_018895589.1        -HCDSSTVSPSTPVSLPRHQSQSDEVDGP--MQRP------------------RPATRNL  143
Aluc_HE_KAF6204998.1          ------PLVRKSSLVPQRRRRRR--AEKP--LRRYAR---------------IQT-----  126
Ofas_HE_GCXY01041673.1        ------PLSRKPPVPPPRRRRRR--SDAK--HHSNLQ---------------EQQQSHQQ  123
Hhal_HE_XP_014287364.1        -------EGRRPPVPPPRRRRRR--CPKA--QHQLD----------------RQ------  95


Clec_HE_XP_014239966.1        ------------------------------EKHPQEMPQQHTLSLGIELRHVEEHTYQS-  102
Btac_HE_XP_018898908.1        GPEPLPRRLGPGSATTKFPSKDADSPSSGPAPGKPHTRHKRWTQVGEELRGIAEDFRSNL  206
Lstr_HE_RZF36208.1            RCSSLRR-------------KAPCCPASLKFSHSLKTSAENWVMVGEELRKIAEDFRSKT  198
Nlug_HE_XP_022189933.1        RCSSLRR--------------KPCCPASLKFSHSLKTSAENWVMVGEELRKIAEDFRTKS  203
Btac_HE_XP_018895589.1        NKRSLSP-------------K-RYLSKLPHSSMKTLRPQKKWVHVGEELRSIAEDFRSTF  189
Aluc_HE_KAF6204998.1          ------------------------TPYDDLRAPPQPVAASSWLNVGIELRQIAEEITFIS  162
Ofas_HE_GCXY01041673.1        QHHSLQH-------------RQESAAARADSAIADLQQGPSWLNVGIELRQIAEEFRSAQ  170
Hhal_HE_XP_014287364.1        -DNSVM------------------------HQDPTPQQGPSWLNVGIELRQIAEEFRSTQ  130
                                                                          :* *** : *.
```

```
Clec_HE_XP_014239966.1      ---------------------VGQTKKRRRESKTSSFFSLLLPTPLPGSIWTTVVIILVGW  141
Btac_HE_XP_018898908.1      S--------------------VRSSRHLPMELKASHLFSLLIPATM-GPVWTTVILLIFP  245
Lstr_HE_RZF36208.1          TTTTTT---SRSDSSQLKHGAPDLPRTKRVELKASSLLSLLVPAPFCGSVWTTVIIIVGW  255
Nlug_HE_XP_022189933.1      TSTTSTTSRPTTDSHRSKCEAPDLPRTKRVELKASSLLSLLVPAPFCGSVWTTVIIIVGW  263
Btac_HE_XP_018895589.1      QKSPLN--------------KTEHSRSPMPEMKASSLFSLLVPSPLGKSVWTTIIFIVGW  235
Aluc_HE_KAF6204998.1        RNG------------------NSSTAKRRSEIKASSLFSLLVPAPVTGTFWTTVIILVGW  204
Ofas_HE_GCXY01041673.1      I--------------------DGSEEKSRKEIKASSLFSLLVPAPLTGSIWTTVIILVGW  210
Hhal_HE_XP_014287364.1      I--------------------GDGVEKKRREIKASSLFSLLVPAPLTGSIWTTVIILVGW  170
                                                          * *:* ::***:*: .   .***:*:::

Clec_HE_XP_014239966.1      RILTRQR---       148
Btac_HE_XP_018898908.1      VIILSSKNNR       255
Lstr_HE_RZF36208.1          KLLMRHNR--       263
Nlug_HE_XP_022189933.1      KLLMRHNR--       271
Btac_HE_XP_018895589.1      RLITRLQ---       242
Aluc_HE_KAF6204998.1        RILSKQR---       211
Ofas_HE_GCXY01041673.1      RILARNR---       217
Hhal_HE_XP_014287364.1      RILARNR---       177
                             ::   .
```
