# Supplementary material for: Deep Conservation of Hid-Like RHG Gene Family Homologs in Winged Insects Revealed by “Taxon Hopping” BLAST
Source: Insects. 2021 Oct 21;12(11):957. doi: 10.3390/insects12110957 (PMC8620624; doi:10.3390/insects12110957)
Supplement: Supplementary file 1 [file insects-12-00957-s001.zip › Supplementary data file S8_ MSA of Aphidoidea RHG protein sequences.pdf]

```
Cced_HEA_VVC35986          MAVPFDFDEIHPE--GNHFTVIQNLLREMLTLLRWLPWTASGNPLPNSTLDNPFVPPYLI 58
Sfla_HEA_XP_025413012      MAVPFRVEG---DSEDHSYLRIENLLKELLTLLRWMPWTTVTGMPTATA--VIDNS---- 51
Apis_HEA_XP_001950202      MAVPFRM-EPDPEVMRRHYLRIQDLLIQLMVQLRQLPSVMIGNSARPSVINYHDLS---- 55
Agos_XP_027846023          MAVPFRPPSHNSDTARDHYLRIENLLRELLTLLSRMPSWMSGDLSSYSNNGINISP---- 56
Acra_HEA_KAF0773171        MAIPFRPPSHNSDTARDHYLRIENLLRELLTLLSRMPSWMNGDLSRYSNNGINVSP---- 56
Agly_HEA_KAE9536444        MAVPFRPPSHNSDTARDHYLRIENLLRELLTLLSRMPSWMNGDLSSYSNNGINISP---- 56
Mper_HEA_XP_022175586      MAIPFVMPEPDPDTPMAHYLRIQNLLRELLTLLSRMPSRMIGDPTNYPYTNISIFS---- 56
Dnox_HEA_XP_015379466      MAIPFVMPEPDPDTPMAHYLRIQNLLRELLTLLRRMPSRIIGDPSNYPYTNISLFS---- 56
Rmai_HEA_XP_026810777      MAIPFNPPSSNPDATRDHYLRIQNLLKELLTLLRRMPSWMIGDQ----YANINMAS---- 52
Msac_HEA_XP_025190335      MAIPFDPPSPNPDTARDHYLQIQNLLKELLTLLRRMPSWMIGDQHNYPYTNINIST---- 56
                           **:**       :     :  *::** :::. *  :*     .

Cced_HEA_VVC35986          QDFPVIQRLMEGVERVRQQVEREDLREQQHQIHLERLRQLRQRAH--QIRQQERQQEREQ 116
Sfla_HEA_XP_025413012      QA----TF-ML---QRRQTAGNRQQSARGLVHHHRRLATAT------------AET-HNR 90
Apis_HEA_XP_001950202      ---PQPDPLEE---RRRPWIQQRQSSE-----HRRRMREEQ------------------- 85
Agos_XP_027846023          QTVPPAQSLEE---RDRLWVQQQQHSIR--MFHRRRLRERQQQERREQQQVVEEEQ-ESQ 110
Acra_HEA_KAF0773171        QTVPPAQSLEE---RDRLWVQQQQHSIR--MFHRRRLRERQQQERREQQQVVEEEQ-ESQ 110
Agly_HEA_KAE9536444        QTVPPAQSLEE---RDRLWVQQQQHSIR--MFHRRRLRERQQQERREQQQVVEEEQ-ESQ 110
Mper_HEA_XP_022175586      SGPPPYSE-EV---RDRLWAQQQQASAR--AFNRRCVRERLQQR-----------Q---- 95
Dnox_HEA_XP_015379466      SDPPPYSE-EV---RDRLWVQQQQSSAR--AFHRRRLRE----R-----------Q---- 91
Rmai_HEA_XP_026810777      QTPLTYLQEEE---NNRLWVQQQQRSMR--AFHRRRLRERQHQERRAQQQELERQQ---- 103
Msac_HEA_XP_025190335      QTPSPYSA-EE---SNRLWVQQQQHSMR--LFHRRRLRERQQE-RREQQQELERQQ---- 105
                                           *    ..:        : . :

Cced_HEA_VVC35986          RRQQQQPGFLPDLVASSVAIPGPAPRLEPDLIRRPERQPAPEPQPEPEPAP--------- 167
Sfla_HEA_XP_025413012      SEGQQQQLPLPDLITSTMSSAPPPPPP--------------------------------- 117
Apis_HEA_XP_001950202      QRSEEQPPLLPDLVASCSQSAGPSHSAGPSHSVGPSHSAGPSHLAGPSHSAGPSHSAGPS 145
Agos_XP_027846023          QHAEQQQVLLPDLVTSCGPTVRQQPPP--------------------------------- 137
Acra_HEA_KAF0773171        QHGEQQQVLLPDLVSSCGPTVRQQPPP--------------------------------- 137
Agly_HEA_KAE9536444        QHVEQQQVLLPDLVTSCGPTVRQQPPP--------------------------------- 137
Mper_HEA_XP_022175586      QQRQQQQSLLPDLVTSCQQSANPPPPP--------------------------------- 122
Dnox_HEA_XP_015379466      QQREQQQPLLPDLVTSCGQAANPPPPP--------------------------------- 118
Rmai_HEA_XP_026810777      QSE-QQRPLLPDLVSSCGPSPRQLPPP--------------------------------- 129
Msac_HEA_XP_025190335      QSDQQQQLLLPDLVSSCGPTSRQPPPP--------------------------------- 132
                               :*   ****::*
```

```
Cced_HEA_VVC35986            -----------------------------------------APAPAPDPVPRSPPQPR-- 184
Sfla_HEA_XP_025413012        --------------------------------------------------PPPPPQP--- 124
Apis_HEA_XP_001950202        HSAGPSHLAGPSHSAGPSQSAGPLQSAGPSQSASPPQSDSSLQSDSPPQSDNPPPQQQLQ 205
Agos_XP_027846023            ----------------------------------------------PPPPPSSQ---QRP 148
Acra_HEA_KAF0773171          ----------------------------------------------PPPPPSSQ---QRS 148
Agly_HEA_KAE9536444          ----------------------------------------------PPPPPSSQ---QRP 148
Mper_HEA_XP_022175586        ----------------------------------------------PPPPPPSPPQQQLQ 136
Dnox_HEA_XP_015379466        ----------------------------------------------PPPPPPSPPQQQLQ 132
Rmai_HEA_XP_026810777        ----------------------------------------------PPPPPSAPLQQQQQ 143
Msac_HEA_XP_025190335        ----------------------------------------------PPPPQ--------Q 138


Cced_HEA_VVC35986            -----GA---YRRKKHVKVESVRIQMCPSCSLNGGTFTAIINTLSTDQQR--E------- 227
Sfla_HEA_XP_025413012        ----------SADKKPTKKILLEVELCPECYYDHQ-KFKVVAANRRDKSNDGLPLSRRHR 173
Apis_HEA_XP_001950202        AQQQTAV---EGEDKIAGAVKLQVAVCPTCYYGKERKLKVIAIAKLDENG--NPI----- 255
Agos_XP_027846023            EQQHQSAVD---DDKFVDAVKLQVAVCPTCYYGKERKLKVIGVAKLDGNG--KPI----- 198
Acra_HEA_KAF0773171          E-QHQSAVD---DDKFVDAVKLQVAVCPTCYYGKERKLKVIGVAKLDGDG--KPI----- 197
Agly_HEA_KAE9536444          E-QHQSAAD---DDKFVDAVKLQVAVCPTCYYGKERKLKVIGVAKLDGNG--KPI----- 197
Mper_HEA_XP_022175586        AQQ-QSAEEEDIEDKIARAVKLQVAVCPTCYYSEERKLKVIAVAKLDENG--NPI----- 188
Dnox_HEA_XP_015379466        AQQ-LSAVD---EDKIGRAVMLQVAVCPTCYYSKEGKLKVIAVAKLDENG--KPI----- 181
Rmai_HEA_XP_026810777        PEQQQSTVD---DDKFVGAVKLQVAVCPTCYYGKERKLKVIAVAKLDENG--KPI----- 193
Msac_HEA_XP_025190335        SVQRQSAVD---DDKFLGAVKLQVALCPTCYYGKERKLKVIAIAKLDENG--KPI----- 188
                                          .*      :.: :** *  .      ::     * .

Cced_HEA_VVC35986            -------PTFDDDDEED-----GYCWEIKRRLKSLFQPSNIDSSILTAAMVIIGWKMFIK 275
Sfla_HEA_XP_025413012        STGIADRRSFDNDAEESVVVVKKS--NVKQRLMSLLTPSKIDSSILTAVFVIIGWKMFIK 231
Apis_HEA_XP_001950202        ------RWTN-RDDEEPVVFENKSSK-VKRRIMSLLQPSHIESSVLTAMFVIIGWKMFIK 307
Agos_XP_027846023            ------RWSTHREVSTYIYFYTTCSFSAQPRVSSNLYQLRXTKRYLLSFYFI-------- 244
Acra_HEA_KAF0773171          ------RWNTHRDDEELLVIEDKSTK-VKRRIMSLLQPSKIESSVLTAMFVIIGWKMFIK 250
Agly_HEA_KAE9536444          ------RWSTHREDEELLVIEDKSTK-VKRRIMSLLQPSKIESSVLTAMFVIIGWKMFIK 250
Mper_HEA_XP_022175586        ------RWTNNRDDEEPMVVENKISK-VKRRIMSLLQPSKIESSVLTAMFVIIGWKMFIK 241
Dnox_HEA_XP_015379466        ------RWTNHRDDEEPVVVENKSSK-VKRRIMSLLQPSKIESSVLTAMFVIIGWKMFIK 234
Rmai_HEA_XP_026810777        ------RWNANRDDEELLVFENKSTK-VKRRIMSLLQPSKIESSVLTAMFVIIGWKMFIK 246
Msac_HEA_XP_025190335        ------RWNTNRDDEELLIVENQSTK-VKRRIMSLLHPSKIESSVLTAMFVIIGWKMFIK 241
                                     .   : .             : *: * :   .  .  * :  .*
```

Cced_HEA_VVC35986	Q	276
Sfla_HEA_XP_025413012	Q	232
Apis_HEA_XP_001950202	Q	308
Agos_XP_027846023	-	244
Acra_HEA_KAF0773171	Q	251
Agly_HEA_KAE9536444	Q	251
Mper_HEA_XP_022175586	Q	242
Dnox_HEA_XP_015379466	Q	235
Rmai_HEA_XP_026810777	Q	247
Msac_HEA_XP_025190335	Q	242
