# Supplementary material for: Deep Conservation of Hid-Like RHG Gene Family Homologs in Winged Insects Revealed by “Taxon Hopping” BLAST
Source: Insects. 2021 Oct 21;12(11):957. doi: 10.3390/insects12110957 (PMC8620624; doi:10.3390/insects12110957)
Supplement: Supplementary file 1 [file insects-12-00957-s001.zip › Supplementary data file S9_ MSA of dictyopteran RHG protein sequences.pdf]

```
Bger_D_PSN4072400          MAIPFRLPTQQETDDNYQILDQLIDQLYHIVRQIILRRGRLVEEAPVPPASTSGYDFSLI   60
Csec_D_XP_02371120400      MAIPFNLPTQQDTEENYDILDQLLEQLYEVMLEILRQRRLRDQL----------YETMLE   50
Znev_D_XP_02192701300      MAIPFNLPTRQETEDNYEIMDQLLEQLYEIVRQIILRRRR--------------------   40
                           *****.***:*:*::**:*:***::***.:: :*: :*

Bger_D_PSN4072400          VYLVKASEIRQVSGNVSGQVL----ATVIRIGACGKRWLVPGRRPTGCRFIPGDRXXXMQ   116
Csec_D_XP_02371120400      -ILRQRRLRDSRSAIPTTLMPHNREAQRRENDAD------GGNGSGEPEPSGSQNQQQA   103
Znev_D_XP_02192701300      ------SRGADNRTGIPA-PEPRNRDTQTRDGGGG------GGNEAGRSDPSVNGNQEQG   87
                                  .  :    :          :  * .  .      * . :*      .

Bger_D_PSN4072400          LNQD-LDLSLPLPDIVLSTGFVPESGPSGSRAPVPPPRRFAPAT---ERPQPPPRRPRRR   172
Csec_D_XP_02371120400      LNRDAMLRVLPQPDVVSNPTT---R-SAQSEVPIPPPRRCTPAGGVRVRPCPPPRRSRRS   159
Znev_D_XP_02192701300      IGNDAMRRIPALPDIVPITDI---RAAAPSDAPVPPPRRGTPEGGVRPRPRRPPRRPRRR   144
                           :..* :      **:*           : * .*:***** :*      **  **** **

Bger_D_PSN4072400          RPVDKRKREDTPEDTRPDQRQRENWLQVGKELRKIADGFRASNSSLQEDGGNRSSTDIEA   232
Csec_D_XP_02371120400      -RSTS--SNEKTSAVPTDNRTRENWQEVGKELRKIADQFCASSS--PEEEGPQTTRHLHN   214
Znev_D_XP_02192701300      TSSCC--CNEKKQGVPNDDRTTENWKEVGKDLRKIADEFCTSSR--LEGEGTQTAPHLHN   200
                                   ::. . .  *:*  *** :***:****** * :*.    *  * ::: .:.

Bger_D_PSN4072400          MKKEQSLLSLLVPAPFRGSLWTAVIFLVGWRILAGRVW   270
Csec_D_XP_02371120400      TKKEESLLSLLLPSRLRGPVWTAVIIIVGWRFLASSGW   252
Znev_D_XP_02192701300      KKKEEGLLSLLLPPRLGGSMWTAVIIILVGWRFLASSGW   238
                            ***:.*****:*  : * :*****:*****:**.  *
```
